# Single‐cell transcriptomics reveals antigen‐presenting capacity and therapeutic resistance potential of immunomodulatory endothelial cells in colorectal cancer

**DOI:** 10.1002/iid3.1311

**Published:** 2024-06-14

**Authors:** Jingyi Wen

**Affiliations:** ^1^ School of Biology and Biological Engineering South China University of Technology Guangzhou Guangdong China

**Keywords:** angiogenesis, antiangiogenic therapies, antigen presentation, colorectal cancer, immunomodulatory, tumor endothelial cells

## Abstract

**Background:**

The heterogeneity of tumor endothelial cells (TECs) hinders the efficacy of antiangiogenic therapies (AATs). Only a small percentage of angiogenic TECs are considered effective targets for AATs. Immunomodulatory ECs (IMECs), as a newly focused functional subgroup of endothelial cells (ECs), are being evaluated for their ability to regulate tumor immune balance and influence existing AATs.

**Methods:**

Based on single‐cell transcriptome data from colorectal cancer in a publicly available database, we conducted a wide array of bioinformatic approaches to study EC subsets that meet the IMECs definition. Our investigation encompassed the gene expression signatures of these subsets, cellular composition differences, cell–cell interactions.

**Results:**

Two subsets that meet the IMECs definition were found in tumors and para‐cancerous tissues. Combined with the results of gene ontological analysis and interaction with CD4^+^ T cells, we found that IMECs can present MHC‐II antigens to mature CD4^+^ T cells. There were differences in the level of interaction between IMECs and different types of mature CD4^+^ T cell subsets. In addition, IMEC subsets had different expression levels of angiogenesis related genes. The angiogenesis score of IMECs decreased after patients received immunotherapy. IMEC subsets do not depend on a single proangiogenic receptor and are involved in regulating angiogenesis, which may reduce the efficacy of AATs. The adverse effects of specific IMEC subsets on AATs were validated in the RNA‐seq dataset of the bevacizumab treatment group.

**Conclusion:**

Our study suggests the potential MHC‐II antigen presentation capacity of IMECs and the enhanced angiogenesis characteristics within tumors. The function of IMECs in the vascular network may have a potentially adverse effect on AATs. Controlling the functional properties of IMECs may be a new angle for tumor therapy.

## Graphical abstract

We found that Immunomodulatory ECs do not present MHC‐II antigens to mature CD4 + T cells with the same intensity. And we found the potential angiogenesis effects of specific Immunomodulatory EC subsets within tumors

## 1 INTRODUCTION

In addition to building a functional vascular network,^1^ vascular endothelial cells (vECs) also play special roles in immune regulation, including immune cell recruitment and antigen presentation.^2^ Based on the involvement of vECs in abnormal angiogenesis within tumors, antiangiogenic therapies (AATs) have been introduced into tumor therapy and has been shown to delay tumor progression in a variety of cancer therapies.^3^ However, AATs are less effective than expected, including drug resistance^3^ and limitations in cancer types.^4^, ^5^ For colorectal cancer (CRC), AATs do not provide clinical benefits for adjuvant treatment of CRC compared with metastatic CRC.^6^ One possible reason is that the phenotype and function of tumor endothelial cells (TECs) are heterogeneous,^7^ providing TECs with a potential escape mechanism for AATs.

Through single‐cell sequencing (scRNA‐seq) technology, it has been confirmed that there are multiple endothelial cell (EC) subsets in tumor tissues. Different subsets have different functional priorities, which reflects the heterogeneity of TECs.^7^ Interestingly, in lung cancer, tip TECs (the target subpopulation of AATs) make up only <10% of all TECs.^8^ In CRC, the heterogeneity of TECs has been preliminarily characterized, vECs can be divided into different subsets, each respectively exhibiting phenotypic characteristics specific to angiogenesis, proliferation, and immunomodulation.^9^, ^10^ Through ECs with immunophenotypes, tumor immunity may be associated with abnormal angiogenesis^11^ targeted by AATs. Therefore, in addition to the classical angiogenesis subgroups, TECs with immunomodulation characteristics may also be of concern.

Recently, it has been suggested that EC subtypes with the gene signatures indicative of phagocytosis or scavenging, antigen presentation and immune cell recruitment^2^ can be collectively referred to as immunomodulatory ECs (IMECs). The characteristics of IMEC phenotype exhibit varying degrees of organ dependency, disease dependency, and treatment dependency. For example, IMECs exhibit capillary signatures in the lungs and vein signatures in the breasts.^8^, ^12^ The phenotypic and functional characteristics of IMECs in the colon and rectum also deserve attention to determine whether IMECs have the potential to be targeted by novel AATs. Meanwhile, as a type of tumor vECs, the relationship between IMECs and existing AATs needs further research, which will determine whether IMECs are related to the therapeutic resistance of existing AATs. Unfortunately, due to ECs only constitute a small fraction of tumor tissues, it is difficult to study specific EC phenotypes.^7^, ^13^ Integrating decentralized datasets could be an important way to address this situation.^14^

In this study, four CRC scRNA‐seq datasets were integrated to distinguish EC subsets within stromal cells and validated using an independent scRNA‐seq validation dataset. Two subpopulations of TECs (EC‐ACKR1 and EC‐KDR‐IGFBP3) were found to have the characteristics of IMEC and can present MHC‐II antigens to mature CD4^+^ T cells. Besides, tumor‐associated IMECs have different expression levels of angiogenesis related genes and may have the ability to evade conventional AATs. Deconvolution analysis identifies a higher proportion of specific IMEC subgroups among patients who were refractory to bevacizumab treatment.

## 2 MATERIALS AND METHODS

### 2.1 Data processing and TEC annotation

The scRNA‐seq data were obtained from the GEO database with access numbers GSE132465,^9^ GSE144735,^9^ GSE178341,^10^ and GSE188711.^15^ We extracted primary CRC tissue cells from 97 patients (a total of 99 samples). Genes and cells that meet the following screening criteria will be retained: (i) A gene was expressed in at least 3 cells; (ii) the number of detected genes was above 500 and below 6000; (iii) the mitochondrial gene content was less than 20%. Finally, 239,163 cells were retained. The R package Seurat (version 4.2.1) was used for clustering of all cells.^16^ To identify major cell types, 2500 highly variable genes were selected for the initial clustering. The top 50 principal components (PCs) were calculated using the “RunPCA” function. Harmony (version 0.1.1) was used to integrate the data to remove batch effects.^17^ Then, the resolution parameter in FindClusters was set to 0.5, and the resulting clusters were visualized using “UMAP.”

Each cell subpopulation was annotated with well‐known marker genes, and stromal cells were extracted for secondary clustering (lymphocytes: CD3D, CD8A, CD4, FOXP3, TRDC, NKG7, CD79A, MS4A1, IGHG4; myeloid cells: CD14, FCGR3A, CD68, CD163, CD1C, LAMP3, TPSAB1, CSF3R, S100A8; stromal cells: PECAM1, COL1A1, VWF; epithelial cells: EPCAM) (Table S1). In the subclustering, 2000 highly variable genes were selected. The top 50 PCs were calculated using the “RunPCA” function. Harmony was used to remove batch effects. Then, the resolution parameter in FindClusters was set to 0.4, and “UMAP” was performed on stromal cells. A total of 14,117 cells were divided into 19 clusters. The marker genes used in this study^7^, ^18^ include SELE, ACKR1, VWF, CD74, PECAM1, ESM1, HES1, CD34, KDR, INSR, FLT1, PODXL, IGFBP3, CLDN5, PTPRB, TM4SF1, GJA5, CXCL12, ID1, PLCG2, MCAM, PCLAF, UBE2C, TK1, STMN1, CENPK, FABP4, PROX1, MMRN1, TFF3, CCL21, LYVE1, COLEC12 (Table S2).

### 2.2 Data processing and corresponding subpopulations annotation in paracancer tissues

The scRNA‐seq data of paracancer tissues were obtained from the GEO database with access numbers GSE132465,^9^ GSE144735,^9^ and GSE178341.^10^ A total of 52 normal tissue samples (52 patients) underwent the same standard of data quality control and batch effect removal as cancer tissue. Finally, 83,649 cells were retained. During the subclustering procedure, the same parameters as cancer tissue were utilized, and 11,521 stromal cells were obtained. Dotplot function was used to evaluate the expression of stromal cell marker gene in the paracancer tissues, and EC subtypes were then identified.

### 2.3 Data processing and corresponding subpopulations annotation in validation dataset

The validation dataset was obtained from the GEO database with access numbers GSE205506,^19^ encompassed 40 samples derived from 19 CRC patients treated with the neoadjuvant PD‐1 blockade. This dataset includes both tumor and normal samples, which were integrated during our validation process. Genes and cells that meet the following screening criteria will be retained: (i) A gene was expressed in at least 3 cells; (ii) the number of detected genes was above 500 and below 6000; (iii) the mitochondrial gene content was less than 50%. During annotation, the validation set adopted the same marker genes and clustering parameters as the original dataset, resulting in 13,916 ECs.

### 2.4 Dynamic evolution analysis of TECs

VECTOR was used to infer the differentiation direction of vECs.^20^ In the subclustering, top 150 PCs were calculated using the “RunPCA” function, and the remaining clustering parameters were unchanged (the clustering results were only used for dynamic evolution analysis). Four types of vECs can still be distinguished in the new cluster. Cell differentiation tracks are mapped to the UMAP plot. In addition, the R package Slingshot (version 2.6.0) was utilized to validate the trajectory analysis conducted using VECTOR.^21^

### 2.5 Expression levels of MHC‐II genes and co‐stimulatory molecules

Using the R package Seurat, we visualized the expression levels of MHC‐II genes (HLA‐DRA, HLA‐DRB1, HLA‐DPA1, HLA‐DPB1, HLA‐DQB1, HLA‐DMA), co‐stimulatory molecules (CD80 and CD86), and cell adhesion molecules (ICAM1 and ICAM2).

### 2.6 Genetic ontological (GO) analysis of tumor vECs

The FindAllMarkers function was used to identify differentially expressed genes (DEGs) with log2FC >1 and *p* < .05. Then the gene symbol was converted into ENTREZID through the Database for Annotation, Visualization and Integrated Discovery (DAVID)^22^, ^23^ (Tables S3–S6). The R package clusterProfiler (version 4.6.0) was used to conduct Gene Ontological (GO) analysis on DEGs in four types of vECs,^24^ including molecular function, cellular component, and biological process. The function “simplify” was used to combine functional signatures with similar significance, cutoff = 0.7. When more than 10 functional signatures were enriched, the top 10 functional signatures were selected for display.

### 2.7 Lymphocyte clustering and CD4^+^ T cell subgroup identification in tumor tissue

The lymphocyte clusters extracted from the first cluster were reclustered. In the subclustering, 2000 highly variable genes were selected. The top 50 PCs were calculated using the “RunPCA” function. Harmony was used to remove batch effects. Then, the resolution parameter in FindClusters was set to 1.5, and “UMAP” was performed on lymphocytes. A total of 113,485 cells were divided into 32 clusters. Marker genes used to identify CD4^+^ T cells include^18^, ^25^, ^26^, ^27^: TCF7, MAL, CCR7, WDR74, CXCL13, PDCD1, CD4, CD8A, RTKN2, FOXP3, ENTPD1, CTLA4, IL2RA, TNFRSF18, SELL, GPR183, CCR6, CD69. Using the same annotation parameters as the original dataset, the validation dataset obtained 5574 antigen‐experienced CD4^+^ T cells.

### 2.8 EC—CD4^+^ T cell ligand pair analysis

The R package CellChat (version 1.6.1) was used to analyze the “Cell‐Cell Contact” interactions between TECs and CD4^+^ T cells,^28^ visualized by the netVisual_bubble function. Then, the network centrality scores of “MHC‐II” were computed and visualized.

### 2.9 Comparison of IMECs transcriptional activity between tumor and paracancer tissues

EC‐ACKR1 and EC‐KDR‐IGFBP3 were extracted and combined from cancer tissue group and paracancer tissue group. The function VlnPlot in R package Seurat (version 4.2.1) was used to visualize the expression of MHC‐II genes. Subsequently, we used the FindMarkers function to identify DEGs between cancer tissue and paracancer tissue with screening thresholds of |log2FC| >0.25 and *p* < .05, and the results were visualized by EnhancedVolcano package (version 1.16.0).^29^ For the obtained genes, the gene symbol was converted into ENTREZID through DAVID (Table S7). The R package clusterProfiler (version 4.6.0) was used to conduct GO analysis on the upregulated and downregulated marker genes of the two subpopulations (EC‐IGFBP3 and EC‐ACKR1), respectively. The function “simplify” was used to combine functional signatures with similar significance, cutoff = 0.7. When more than 10 functional signatures were enriched, the top 10 functional signatures were selected for display.

### 2.10 Gene module enrichment analysis

To assess potential dissociation‐induced bias, we calculate dissociation scores for various vEC subsets within each sample based on the expression levels of immediate‐early genes (FOSB, FOS, JUN, JUNB, JUND, ATF3, and EGR1) and heat shock proteins (HSPA1A, HSPA1B, HSP90AB1, HSPA8, HSPB1).^30^ To investigate the antigen presentation functions of MHC‐II in IMECs, we calculated the MHC‐II signature scores for each cell, employing genes specific to the MHC class II family, including HLA‐DRB5, HLA‐DRB1, HLA‐DRA, HLA‐DQB1, HLA‐DQA1, and HLA‐DPA1. VEGFR signature score is based on genes FLT1, FLT4, KDR, angiogenic activity signature score is based on selected differential genes in the entry (GO:0045765, regulation of angiogenesis) that were enriched in both tumor‐associated IMEC subpopulations (Table S7). All the above calculations were performed using the AddModuleScore function from the R package Seurat (version 4.2.1). In addition, the function VlnPlot in R package Seurat (version 4.2.1) was used to visualize the expression levels of proangiogenic receptors genes (FGFR1, FGFR2, FLT1, KDR, FLT4, PDGFRA, PDGFRB, KIT, MET).

### 2.11 EC ligand pair analysis

The R package CellChat (version 1.6.1) was used to analyze the “Cell‐Cell Contact” interactions between IMECs and other ECs, visualized using the netVisual_bubble function. Then, the comprehensive network centrality scores of the detected path were computed and visualized.

### 2.12 Deconvolution analysis and evaluation of drug response differences

The above‐mentioned extracted stromal cells were re‐annotated and classified into five subgroups of typical ECs (EC‐KDR‐IGFBP3, EC‐ACKR1, EC‐KDR‐ESM1, EC‐STMN1, EC‐TFF3) and the other four subgroups of stromal cells: fibroblasts (Fb), mural cells (Mu), smooth muscle cells, EC‐Mu co‐expressing cell populations (EC‐Mu). Converted the commented Single cell reference matrix file into Signature matrix file by using the CIBERSORTx ^31^ (Table S8).

Data on response to bevacizumab therapy were obtained from the GEO database with access numbers GSE19862 and GSE19860. The study included 12 patients who responded to the therapy and 14 who did not. Using CIBERSORTx for gene‐chip data deconvolution, we deduced the proportion of stromal cells accounted for by specific EC subsets in these patients (Table S9). “Disable quantile normalization” was selected, and the remaining parameters involved were the default parameters of the CIBERSORTx. The genes involved in the deconvolution process were all stromal cell marker genes obtained in the initial clustering (Table S8).

### 2.13 Statistical analysis

Statistical analyzes in this study were implemented in R (version 4.2.1) and *p* < .05 was considered statistically significant. The detailed code is available from the link of GitHub (https://github.com/wenjingyi-1/001-IMECs).

## 3 RESULTS

### 3.1 The atlas of TECs showed heterogeneity and plasticity

**Figure 1:**
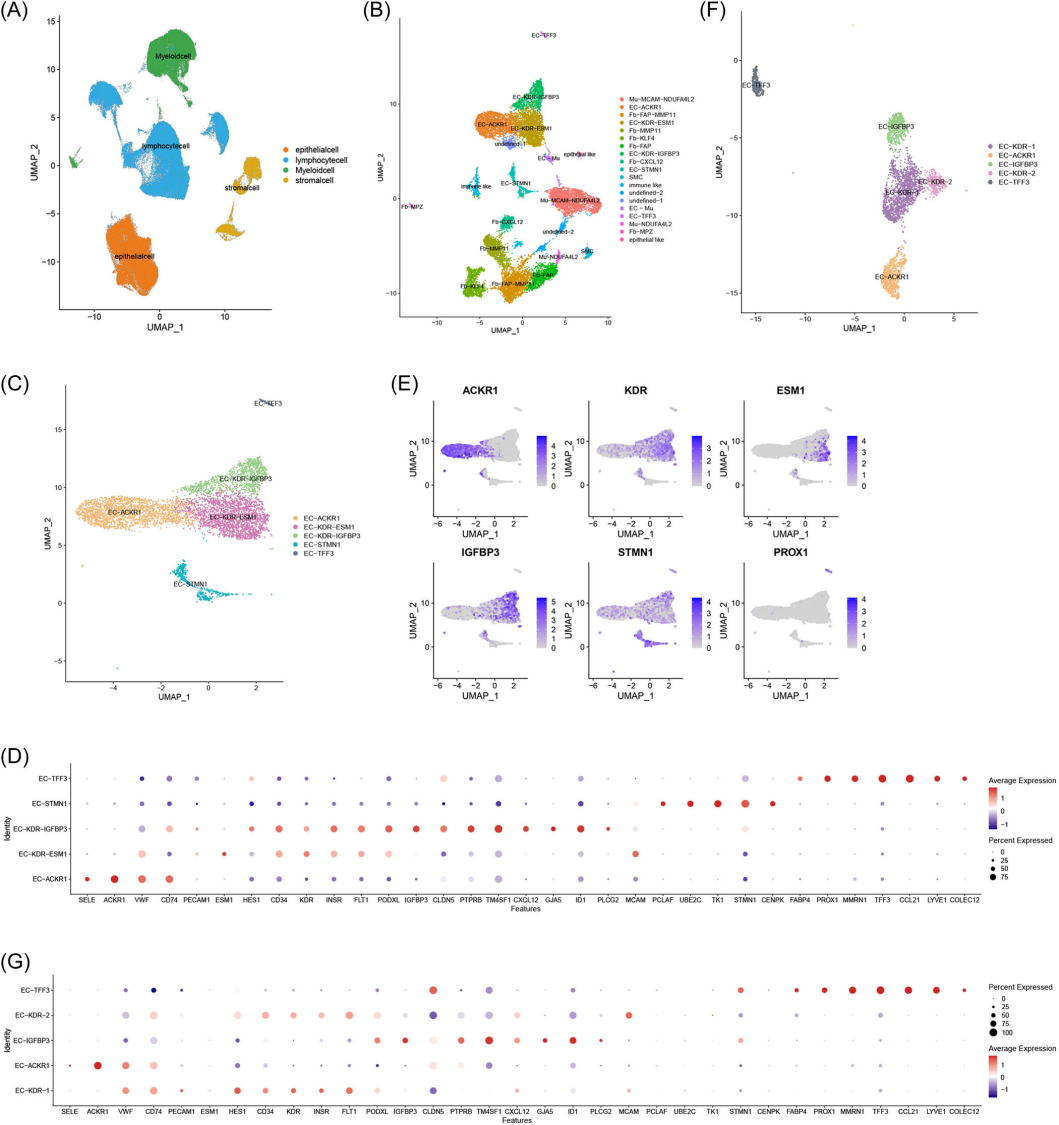
The heterogeneity landscape of endothelial cells. (A) UMAP plot showing tumor microenvironment clusters. Cells are classified into four categories: myeloid cell, lymphocyte, stromal cell, Epithelial cell. (B) UMAP clustering of stromal cells. Please note that two subsets with low marker gene expression cannot be classified. (C) UMAP plot displaying typical EC subsets obtained from the subclustering. (D) Dotplot shows the expression of selected marker genes in TEC subsets. (E) Expression of key marker genes in TECs. (F) UMAP plot displaying EC subsets of paracancer tissue. (G) Dotplot shows the expression of selected marker genes in normal EC subsets. EC, endothelial cell; TECs, tumor endothelial cell.

After initial clustering of 99 untreated primary CRC samples from 97 patients, a total of 239,163 cells were divided into 24 clusters. All clusters were grouped into 4 major cells types (Figure 1A; Figure S1A), and stromal cells were reclustered (Figure 1B; Figure S1B,C).

Stromal cells were annotated using canonical cellular markers from the literature.^7^, ^18^ Five subsets were identified as EC (*n* = 5100 cells) (Figure 1C–E). Specifically, EC‐ACKR1 were identified based on ACKR1, which was associated with venous ECs (Figure 1D,E). The expression profiles of EC‐KDR‐ESM1 and EC‐KDR‐IGFBP3 are similar to some extent, including the proangiogenic receptor family (KDR, INSR, FLT1), highlighting their angiogenic potential. However, EC‐KDR‐IGFBP3 overexpression was observed for IGFBP3, CLDN5 and ID1, while ESM1 expression was relatively weaker (Figure 1D,E). The biological functions of these two clusters may be different. EC‐STMN1 showed high expression of proliferation‐associated genes STMN1, UBE2C and TK1, which are characteristics of proliferative ECs (Figure 1D,E). In addition, EC‐TFF3 were identified as lymphatic ECs based on PROX1, CCL21, COLEC12, and TFF3 (Figure 1D,E).

Finally, EC‐ACKR1, EC‐KDR‐ESM1, EC‐KDR‐IGFBP3, EC‐STMN1 were considered to be the vascular‐associated subgroups of TECs studied herein.

By annotating stromal cells within the paracancer tissue,^7^, ^18^ we aimed to identify differences in ECs between tumor tissue and paracancer tissue (*n* = 3419) (Figure 1F,G; Figure S1D–F). Interestingly, the typical proliferative ECs were not found in paracancer tissue (Figure 1G). With the exception of EC‐STMN1, all EC subpopulations in paracancer tissue have some similarity to TECs. Specifically, EC subgroups characterized by high expression of proangiogenic receptors and EC‐ACKR1 with high expression of ACKR1 were also identified in paracancer tissues (Figure 1G). The difference, however, was that ECs with high expression of proangiogenic receptors decreased the expression of key genes used to distinguish them, namely ESM1 and IGFBP3. A unique cluster of ECs was identified with high expression of IGFBP3, CLDN5, ID1 but weak expression of KDR, which closely resembled EC‐KDR‐IGFBP3 (Figure 1G). This suggests phenotypic differences between ECs in the tumor and paracancer tissues, which could be related to differences in the microenvironment properties of the two tissue types.

**Figure 2:**
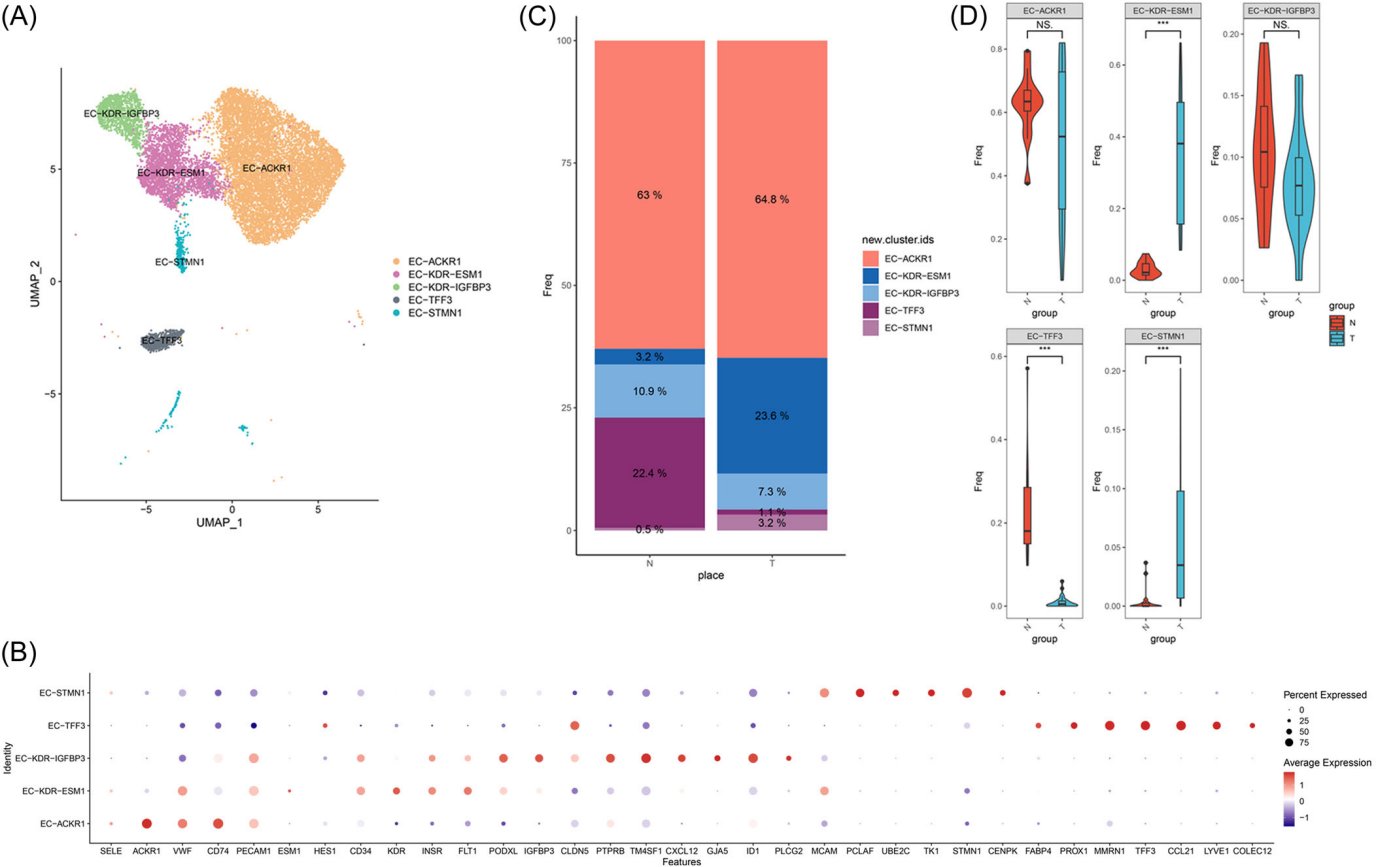
Differences of endothelial cells in tumor tissue and adjacent tissue in validation dataset. (A) UMAP plot displaying EC subsets obtained from the subclustering. (B) Dotplot shows the expression of selected marker genes in EC subsets. (C) Proportion of five EC subtypes represented in bar charts across different tissue types. (D) The proportion of endothelial cell subsets in each sample relative to the total endothelial cells was compared (wilcox test; *p < .05, **p <  .01, ***p < .001). EC, endothelial cell.

For validation, we employed an integrative analysis strategy on a dataset comprising 27 tumor samples and 13 normal samples obtained from 19 patients with CRC undergoing neoadjuvant PD‐1 blockade therapy. The validation dataset's dissociation score gene signature did not differ significantly (Figure S2A,B), thus ruling out potential dissociation‐induced bias in the results. Using the TEC labeling strategy from the original dataset, the validation set of ECs (*n* = 13,916) could also be grouped into the same five categories as the original dataset: EC‐ACKR1, EC‐KDR‐ESM1, EC‐KDR‐IGFBP3, EC‐STMN1, and EC‐TFF3 (Figure 2A,B; Figure S3A,B). In addition, consistent with the observations in original dataset, EC‐STMN1 and EC‐KDR‐ESM1 were primarily detected in tumor tissue with significantly lower percentages observed in paracancer tissue (Figure 2C,D).

**Figure 3:**
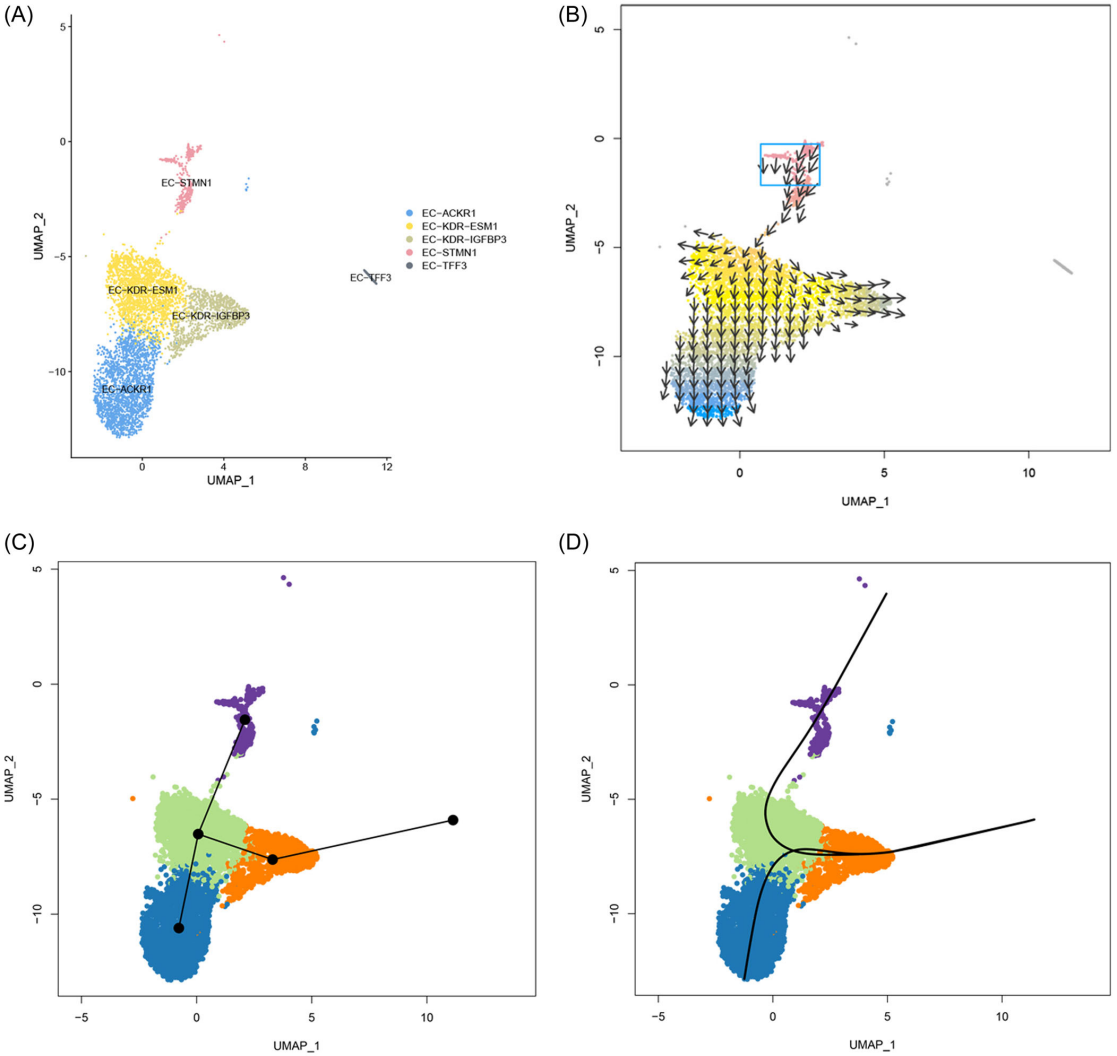
Dynamic evolution analysis of tumor endothelial cells. (A) The positioning of each EC subset. (B) Dynamic evolution trend of TECs derived from the VECTOR. (C, D) Dynamic evolution trend of TECs derived from the Slingshot. TEC, tumor endothelial cell.

VECTOR was used to infer the differentiation direction of vECs (Figure 3A,B). The results showed that proliferative ECs were the origin of tumor vECs development (Figure 3A,B). The proliferative ECs differentiated into EC‐KDR‐ESM1. Later, some EC‐KDR‐ESM1 differentiated into EC‐ACKR1 and EC‐KDR‐IGFBP3 along different pathways, while the remaining EC‐KDR‐ESM1 retained its phenotype. Meanwhile, we utilized Slingshot to validate the trajectory analysis performed using VECTOR. The validation outcomes reinforce our initial findings, indicating that EC‐ACKR1 and EC‐KDR‐IGFBP3 reside in different pathways (Figure 3C,D). The differences in ECs differentiation directions suggest that different EC subgroups may have different functional forms. At the same time, proliferative ECs located in the early stage of differentiation were abundant in the tumor, suggesting the plasticity of vascular development within the tumor.

### 3.2 EC‐ACKR1 and EC‐KDR‐IGFBP3 exhibit phenotypic characteristics of IMECs and have the potential to recruit mature CD4^+^ T cells

**Figure 4:**
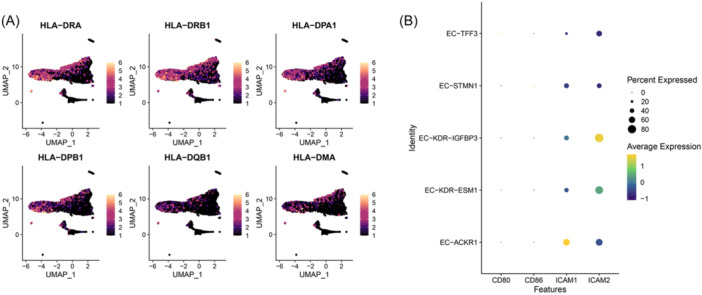
Antigen presenting potential of EC‐ACKR1 and EC‐KDR‐IGFBP3. (A) Expression of MHC class II genes in TECs. (B) Expression of co‐stimulatory molecules and cell adhesion molecules in TECs. TEC, tumor endothelial cell.

IMECs can participate in MHC‐II antigen presentation.^2^, ^32^ To explore the antigen presentation ability of different tumor vEC subsets, we compared the MHC‐II gene expression levels of different EC subsets (Figure 4A). The results revealed that the expression of MHC‐II genes in EC subgroups displayed a distinct subgroup pattern. Specifically, MHC‐II genes were mainly highly expressed in EC‐ACKR1 and EC‐KDR‐IGFBP3, and weakly expressed in other clusters.

In parallel, we examined the expression levels of the classical co‐stimulatory molecules CD80 and CD86, and the cell adhesion molecules ICAM1 and ICAM2 (Figure 4B). The results showed that TECs generally lacked expression of CD80 and CD86, indicating that TECs are not involved in activating naive T cells. ICAM1 can be detected in EC‐ACKR1, and ICAM2 can be detected in EC‐KDR‐IGFBP3, which preliminarily indicates their ability to recruit immune cells.

**Figure 5:**
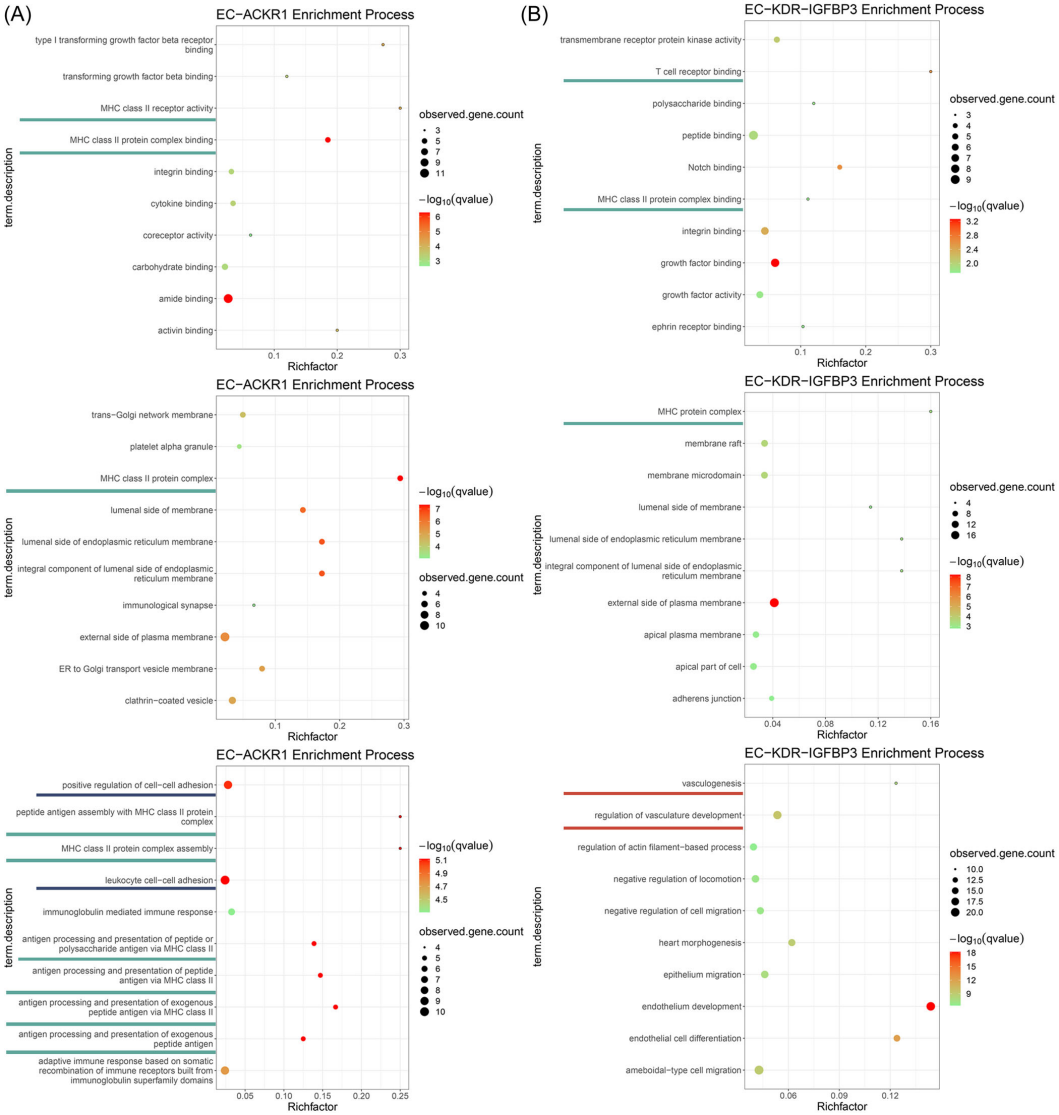
GO analysis of EC‐ACKR1 and EC‐KDR‐IGFBP3, shown from top to bottom as MF, CC, and BP. The biological functions of interest have been outlined, where antigen presentation is represented by the green line, immune cell recruitment by the blue line, and angiogenesis regulation by the red line. (A) Results of EC‐ACKR1. (B) results of EC‐KDR‐IGFBP3. BP, biological process; CC, cellular component; GO, Gene Ontological; MF, molecular function.

GO analysis of marker genes showed the specific biological functions of different tumor vEC subsets (Figure 5A,B; Figure S4A,B). Two subgroups with high expression of MHC‐II genes, EC‐ACKR1 and EC‐KDR‐IGFBP3, were enriched in functions related to the processing and presentation of MHC‐II antigens (Figure 5A,B). EC‐ACKR1 also showed the ability to recruit and activate T cells and leukocytes (Figure 5A). Other EC subgroups were not enriched in functions directly related to MHC‐II antigen presentation (Figure S4A,B). In addition, GO analysis showed that EC‐KDR‐IGFBP3 could regulate angiogenesis, implying its potential impact on tumor angiogenesis (Figure 5B).

**Figure 6:**
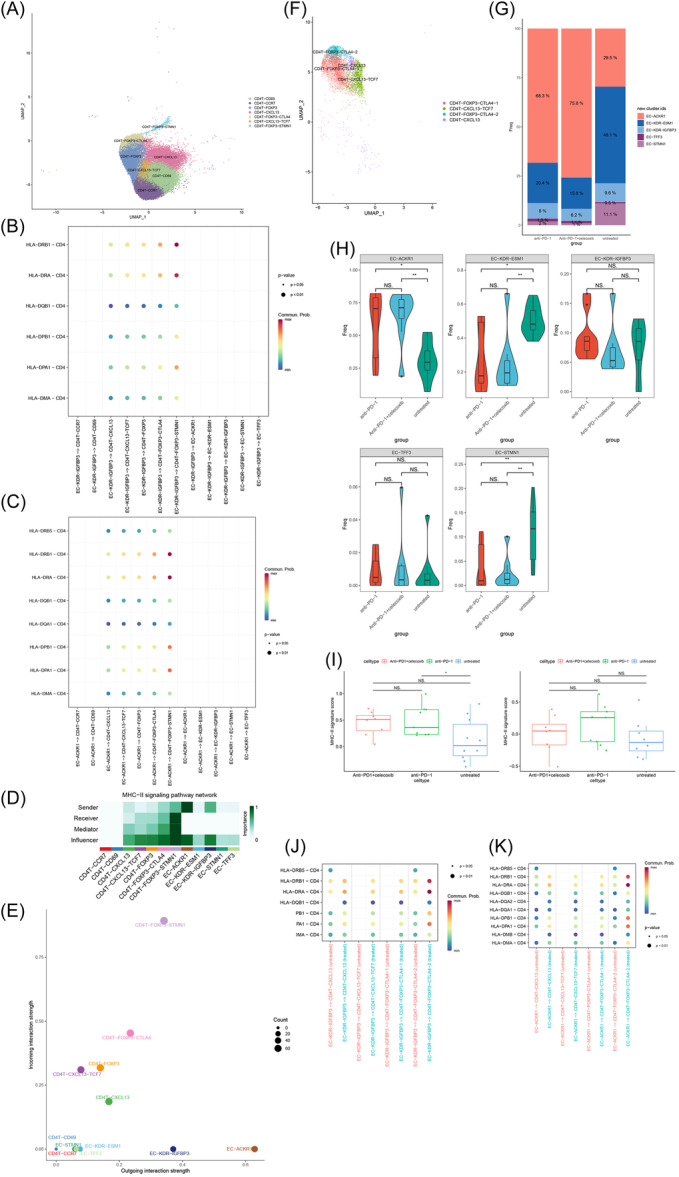
EC‐CD4^+^ T cell interaction and immunotherapy‐induced changes. (A) UMAP clustering of CD4^+^ T cells. (B, C) Bubble map showing MHC‐II interactions between IMECs and CD4^+^ T cells. Large dots indicate statistical significance (*p* < .01, derived from CellChat), while small dots indicate no significance (*p* > .05). (D) network centrality scores of “MHC‐II.” (E) Visualization of MHC‐II interaction strengths between ECs and CD4^+^ T cells in a two‐dimensional space, with a focus on the dominant senders (sources) and receivers (targets). (F) UMAP clustering of antigen‐experienced CD4^+^ T cells in the validation dataset. (G) Proportion of five EC subtypes represented by the bar chart in pre‐ and postimmunotherapy states. (H) The proportion of endothelial cell subsets in each sample relative to the total endothelial cells was compared (wilcox test; *p < .05, **p < .01, ***p < .001). (I) Boxplot of average MHC‐II feature scores for IMECs in patients not treated (*n* = 10), receiving anti‐PD‐1 + celeoxib therapy (*n* = 8), and receiving anti‐PD‐1 therapy (*n* = 9). The left panel displays scores for EC‐ACKR1, while the right panel shows scores for EC‐KDR‐IGFBP3 (wilcox test; *p < .05, **p < .01, ***p < .001). (J, K) Bubble map showing MHC‐II interactions between IMECs and antigen‐experienced CD4^+^ T cells in pre‐ and postimmunotherapy states. EC, endothelial cell; IMEC, immunomodulatory EC.

IMECs, as a subset of antigen‐presenting cells that express MHC‐II molecules, are capable of interacting with CD4^+^ T cells. To further characterize the IMEC subsets within the tumor, we evaluated the interaction between MHC‐II receptors expressed on TECs and MHC‐II ligands expressed on CD4^+^ T cells (Figure 6A–C; Figure S1G). The findings revealed that EC‐ACKR1 and EC‐KDR‐IGFBP3 provide MHC molecules for antigen presentation, and these two subsets can interact with neoantigen‐specific CD4^+^ T cells (CXCL13^+^) and Treg (FOXP3^+^) via MHC‐II ligand pair (Figure 6B,C). No MHC‐II pathway interaction was detected with CD4^+^ T ‐CCR7 and CD4^+^ T ‐CD69 (Figure 6B,C). This finding supports the view that antigen‐presenting ECs primarily activate antigen‐experienced T cells.

Surprisingly, differences were observed in the strength of interactions between the same EC and different CD4^+^ T cell subsets. More specifically, ECs were found to interact more strongly with Treg (FOXP3^+^) compared to neoantigen‐specific CD4^+^ T cells (CXCL13^+^), and display the strongest interaction with the CD4^+^ T ‐FOXP3‐STMN1 (Figure 6B,C). The mechanisms underlying this observation require further investigation.

Furthermore, network centrality scores for MHC‐II were compared across various ECs and CD4^+^ T cells (Figure 6D,E). The results indicate that EC‐ACKR1 and EC‐KDR‐IGFBP3 serve as the core sender in the MHC‐II pathway, while other EC subgroups are not significant contributors. These results further support that the immunomodulatory function of EC is primarily executed by distinct subsets.

In summary, our data demonstrate that EC‐ACKR1 and EC‐KDR‐IGFBP3 possess key characteristics of IMECs, and are involved in antigen presentation processes that specifically drive tumor‐specific immune responses, primarily for the recruitment of functional CD4^+^ T cells within the tumor.

Leveraging the neoadjuvant PD‐1 blockade therapy information from the validation dataset, we examined potential immune function alterations in IMECs within tumors, comparing pre‐ and postimmunotherapy states. Notably, postimmunotherapy, EC‐ACKR1 proportions increased significantly, whereas EC‐KDR‐IGFBP3 proportions remained relatively unchanged (Figure 6G,H). Meanwhile, patients treated with anti‐PD‐1 therapy exhibited a significantly elevated MHC‐II signature score for EC‐ACKR1 (Figure 6I), indicating that EC‐ACKR1 may be a potential immune response subgroup. Additionally, MHC‐II antigen presentation between IEMCs and CD4^+^ T cells was markedly elevated following immunotherapy (Figure 6J,K). Nonetheless, the greatest MHC‐II interaction was identified between IMECs and the Treg subgroup (CD4^+^T‐FOXP3‐CTLA4‐2) (Figure 6J,K).

**Figure 7:**
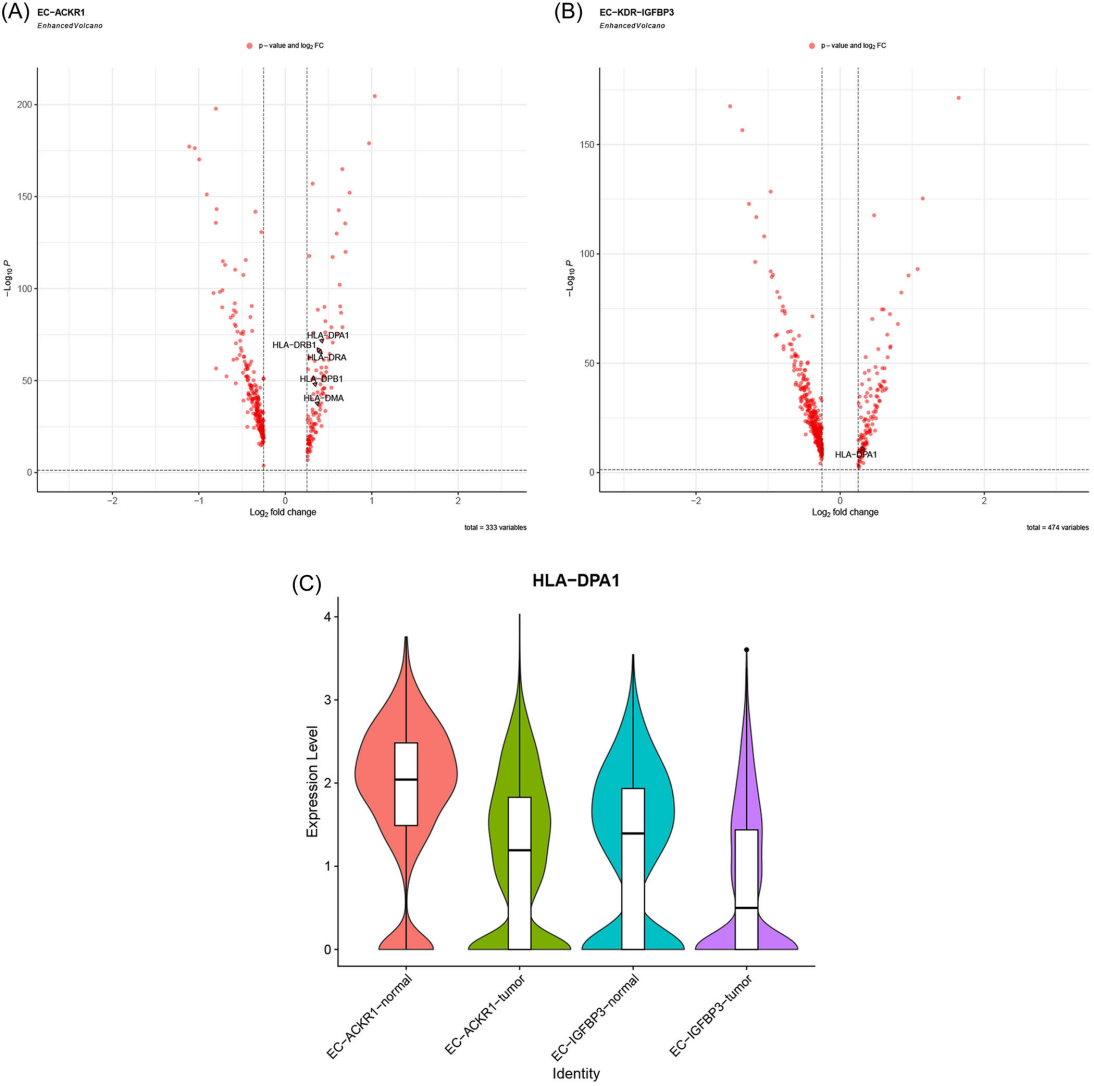
Comparison of IMECs transcriptional activity between tumor and paracancer tissues. (A, B) On the left side of the volcano plot are genes that are downregulated in the paracancer tissue compared to the tumor tissue, while on the right side are genes that are upregulated in the paracancer tissue. Genes of interest are specifically identified (wilcox test; *p* < .05, |log2FC| >0.25). (C) Violin plot specifically portrays the HLA‐DPA1 expression level in distinct subsets of IMECs [the screening threshold in (A) and (B) is satisfied]. EC, endothelial cell; IMEC, immunomodulatory EC.

We compared the transcriptional activity in tumor‐associated IMECs to similar subsets of paracancer tissues. Tumor‐associated IMECs had lower HLA expression compared to IMECs in paracancer tissues (Figure 7A–C). In addition, GO analysis revealed a reduced transcriptional activity of genes involved in MHC‐II antigen processing and presentation (Figures S5 and S6). The downregulation of HLA expression may indicate the impairment of immune regulatory function in tumor‐associated IMECs, and how this change in transcriptional activity will result in changes in IMECs function needs further study.

### 3.3 Based on gene expression differences, IMEC subgroup EC‐KDR‐IGFBP3 exhibits high levels of angiogenesis in the tumor microenvironment

**Figure 8:**
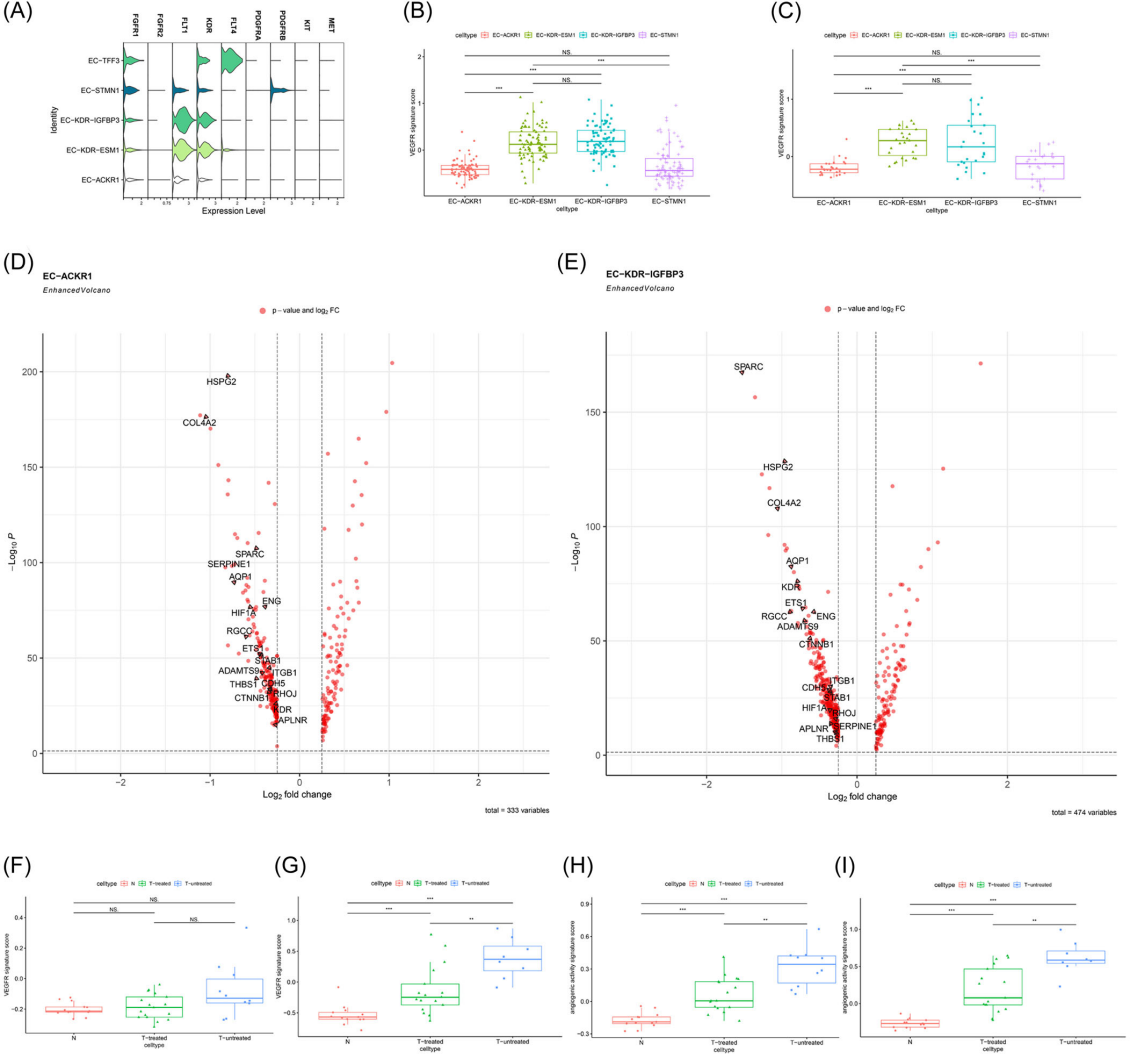
The expression profiles of angiogenesis pathways in distinct IMEC subsets. (A) Violin plot portrays the expression levels of proangiogenic receptors genes. (B) Boxplot of average VEGFR signature scores for different vEC subpopulations in the original dataset. (C) Boxplot of average VEGFR signature scores for different vEC subpopulations in the validation dataset. (D, E) On the left side of the volcano plot are genes that are downregulated in the paracancer tissue compared to the tumor tissue, while on the right side are genes that are upregulated in the paracancer tissue. Genes of interest are specifically identified (wilcox test, *p* < .05, |log2FC| >0.25). (F, G) Boxplot of average VEGFR signature scores for IMECs pre‐ and post‐immunotherapy in validation dataset. (F) EC‐ACKR1 results. (G) EC‐KDR‐IGFBP3 results. (H, I) Boxplot of average angiogenic activity signature scores for IMECs pre‐ and postimmunotherapy in validation dataset. (H) EC‐ACKR1 results. (I) EC‐KDR‐IGFBP3 results [for (B), (C), (F)–(I), wilcox test; *p < .05, **p < .01, ***p < .001]. EC, endothelial cell; IMEC, immunomodulatory EC; vEC, vascular endothelial cell.

The relationship between IMECs, a functional subset of ECs, and AATs needs to be further evaluated. First, we evaluated the expression levels of proangiogenic receptor families (VEGFR, FGFR, PDGFR, c‐Kit, c‐Met) in TECs (Figure 8A). These receptors can serve as binding sites for antiangiogenic small molecule receptor tyrosine kinase inhibitors (TKIS) in the treatment of advanced cancer patients.^33^

The typical angiogenic subgroup EC‐KDR‐ESM1 was used as a control to focus on the receptor gene expression of IMECs. The results revealed weak expression of FGFR (FGFR1, FGFR2), PDGFR (PDGFRA, PDGFRB), KIT and MET in the TECs obtained in this study (Figure 8A). For VEGFR family, the EC‐KDR‐ESM1 subgroup has high expression of FLT and KDR, and the immune‐related EC‐KDR‐IGFBP3 subgroup shows similar expression levels to EC‐KDR‐ESM1 (Figure 8A). In contrast, the EC‐ACKR1 has low expression levels of FLT and KDR (Figure 8A). Furthermore, we compared the VEGFR signature scores among four subgroups of tumor‐associated vECs. As expected, the VEGFR expression levels were notably higher in EC‐KDR‐IGFBP3 and EC‐KDR‐ESM1 when compared to the other subgroups. This was observed both in the original dataset (Figure 8B) and the validation dataset (Figure 8C). It is suggested that EC‐ACKR1 may display weak reactivity towards anti‐VEGFR drugs.

Additionally, VEGFR1 and VEGFR2 can bind to VEGF proteins and activate downstream signaling responses.^34^ They are both highly expressed in the same EC subgroup (EC‐KDR‐IGFBP3, EC‐KDR‐ESM1) (Figure 8A). This suggests that drugs targeting individual VEGF receptors may not be able to completely block downstream signaling pathways, and compensation between pathways may lead to resistance to AATs.

Finally, the results of GO suggest that the transcriptional activity of angiogenesis genes is upregulated in tumor‐associated IMECs compared to IMECs in paracancer tissues (Figures S5 and S6). The angiogenesis‐related genes that are increased in both types of IMEC subsets include VEGFR receptor genes KDR, along with potential proangiogenic genes RHOJ,^35^ ETS1^36^ and HIF1A^37^, ^38^ (Figure 8C,D; Table S7). This suggests that tumor‐associated IMECs display angiogenic behavior.

In the validation set, we further compared the differences in VEGFR signature scores and angiogenic activity signature scores of IMECs between tumor tissue (treated group, *n* = 17; untreated group, *n* = 10) and paracancer tissue (*n* = 13) (Figure 8F–I). The results showed a significant elevation in VEGFR signature scores for EC‐KDR‐IGFBP3 in tumor tissue compared to paracancer tissue (Figure 8G). While EC‐ACKR1 also exhibited some increase, the difference did not reach statistical significance (Figure 8F). Regarding angiogenic activity signature scores, both subgroups of IMECs exhibited a significant increase within the tumor tissue (Figure 8H,I). IMECs may have synergistic or alternative effects with classical angiogenic ECs in tumor tissue, and further validation is necessary.

Interestingly, following immunotherapy, scores of IMECs exhibited a moderate reduction (Figure 8F–I). Although immunotherapy did not reduce their scores to the same level as that of adjacent noncancerous tissues, the potential inhibitory effect of immunotherapy on angiogenic activity is of interest.

In summary, we observed cross‐talk within the angiogenic receptor and functional convergence between different subsets, both of which may represent distinct mechanisms for EC subsets to circumvent AATs.

**Figure 9:**
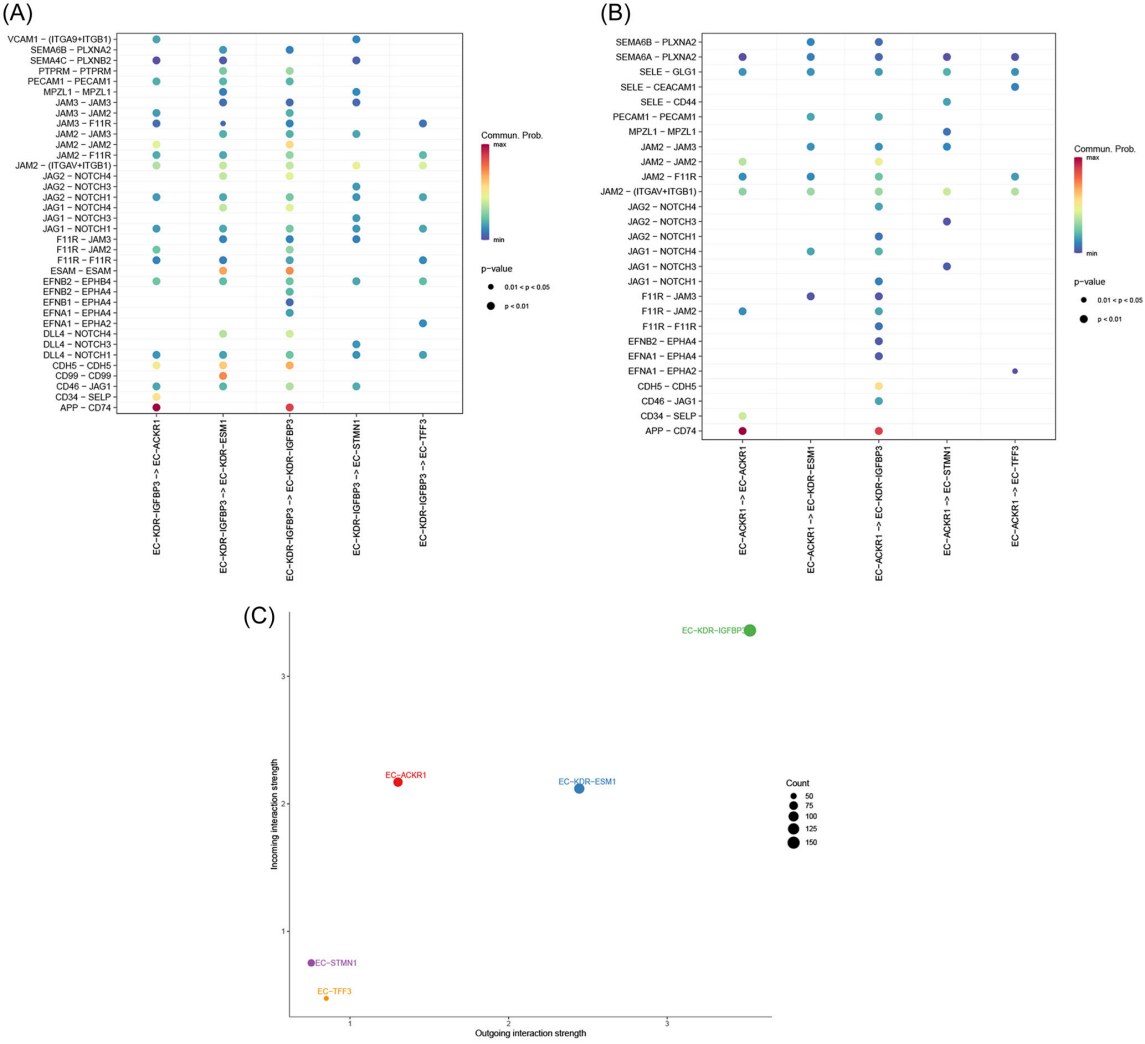
Endothelial cell interactions. (A, B) Bubble map showing comprehensive interactions between IMECs and other ECs. Large dots indicate *p* < .01, while small dots indicate .01 < *p* < .05 (derived from CellChat). (C) Visualization of comprehensive interaction strengths between IMECs and other ECs in a two‐dimensional space, with a focus on the dominant senders (sources) and receivers (targets). EC, endothelial cell; IMEC, immunomodulatory EC.

By analyzing the potential interaction pathways within each EC subgroup, it is found that there are multiple interactive pathways among ECs, some of which have been identified to be involved in vascular regulation (Figure 9A,B). Specifically, through regulation of the NOTCH signaling pathway, ECs can influence the generation of adjacent microvessels^39^, ^40^, ^41^; EC migration and angiogenesis can be also mediated through EphA‐type receptors.^42^, ^43^ The scores of integrated pathways indicate that EC‐KDR‐ESM1, EC‐KDR‐IGFBP3, and EC‐ACKR1 can function as both transmitters and receivers of cell interaction signals, with EC‐KDR‐IGFBP3 having the highest score (Figure 9C). This suggests that these three subgroups may regulate each other and respond to vascular regulatory signals, participating in the formation of a complex angiogenesis regulatory network.

### 3.4 IMECs are not the target cell population for bevacizumab therapy

**Figure 10:**
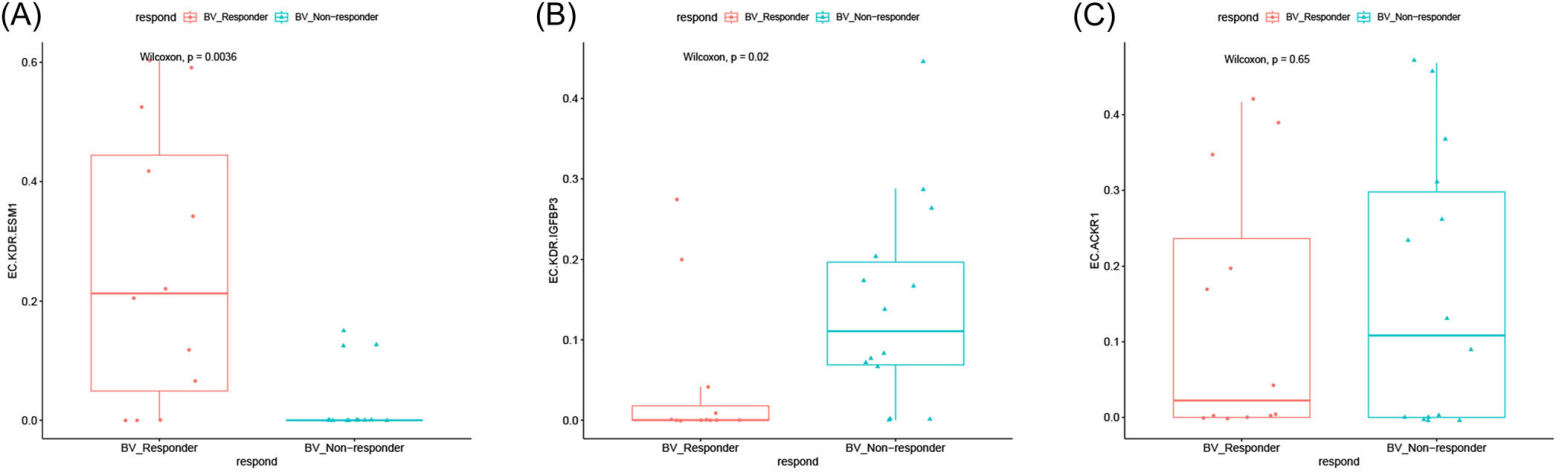
The correlation between distinct subsets of ECs and the response to bevacizumab therapy (wilcox test, *p* < .05 is statistically significant). (A) The level of EC‐KDR‐ESM1 in the responding group versus the nonresponding group. (B) The level of EC‐KDR‐IGFBP3 in the responding group versus the nonresponding group. (C) The level of EC‐ACKR1 in the responding group versus the nonresponding group. EC, endothelial cell.

As IMECs are a cell population with the potential to resist AATs and participate in angiogenesis, it is necessary to further evaluate the impact of IMECs on AATs. Bevacizumab is a prototypical representative within the AATs class of drugs. We employed deconvolution methods to estimate the levels of IMECs in CRC bevacizumab responders (*n* = 12) and CRC bevacizumab nonresponders (*n* = 14) (Figure 10). The results demonstrated that the angiogenic EC subgroup, EC‐KDR‐ESM1, was a responsive subgroup to bevacizumab therapy (*p* < .05). However, EC‐KDR‐IGFBP3 was associated with nonresponse to bevacizumab therapy, and the difference in cell number between the two groups was statistically significant (*p* < .05). Furthermore, no evidence was found in this study to support a role for EC‐ACKR1 in bevacizumab therapy (*p* > .05).

## 4 DISCUSSION

The single‐cell resolution can facilitate our understanding of TECs heterogeneity. Currently, the TECs within almost all cancer types have been profiled using scRNA‐seq.^7^ To investigate specific EC subsets, TECs were obtained from the integrated dataset of primary CRC, and the clustering results were largely consistent with the existing atlas studies.^9^, ^10^ A comparison with adjacent tissues revealed a significant enrichment of proliferative ECs within the tumor,^9^ and the expression patterns of marker genes specific to other ECs were also partially modified. The results of dynamic evolution analysis suggested that proliferative ECs were at an early stage of differentiation, while the other EC subsets followed distinct developmental trajectories. TECs are heterogeneous^44^, ^45^ and plastic^46^ in the tumor microenvironment, including in CRC.

Some TECs act as semi‐professional antigen‐presenting cells in tumor immune regulation.^7^, ^8^ ECs exhibited characteristics of phagocytosis or scavenging, antigen presentation and immune cell recruitment can be referred to as IMECs.^2^ In this study, EC‐ACKR1 and EC‐KDR‐IGFBP3 were found to express MHC‐II genes and cell adhesion molecules, but lacked expression of the T cell activation ligands CD80 and CD86.^47^ Concurrently, GO analysis of these two subsets revealed gene sets associated with antigen processing and presentation. Additionally, EC‐ACKR1 and EC‐KDR‐IGFBP3 were found to engage MHC‐II interactions with neoantigen‐specific CD4^+^ T cells and Tregs, indicating their potential to recruit mature CD4^+^ T cells.^2^ In summary, EC‐ACKR1 and EC‐KDR‐IGFBP3 display specific immunomodulatory functions and are classified as IMECs.

Given that neoantigen‐specific CD4^+^ T cells^48^, ^49^, ^50^ and Tregs^51^, ^52^, ^53^ play distinct roles in tumor immunity, the differential recruitment ability of IMECs towards these two CD4^+^ T cell subsets may impact the delicate balance between antitumor immunity and immunosuppression. In MHC‐II interactions, IMECs engage in stronger interactions with Tregs than with neoantigen‐specific CD4^+^ T cells. Additionally, transcriptional activities of genes involved in antigen presentation and processing are downregulated in IMECs within tumors.^9^, ^54^ Although the recruitment and activation of CD4^+^ T cells are also modulated by the number of cells and the expression patterns of co‐stimulatory molecules,^55^ which involves a complex regulatory process, this nevertheless provides new leads for studying the role of IMECs in tumor immune balance. Significantly, we can control the phenotype of IMECs to improve immune balance, thus potentiate antitumor immune responses, which may offer a promising therapeutic strategy.

The heterogeneity of TECs is an important factor in resistance to AATs, that is, AATs only target part of angiogenic subpopulations.^7^, ^8^ GO analysis has suggested that IMECs also possess the function of regulating angiogenesis. Sensitivity associations between IMEC subsets and AATs need to be evaluated. TKIs are important drugs that target angiogenesis, and currently approved major targets of TKIs include VEGFR, PDGFR, FGFR, c‐Kit, and c‐Met.^33^ In this study, it was difficult to detect the gene expression of FGFR, PDGFR, c‐Kit, and c‐Met in ECs. For the VEGF receptors, the expression level of EC‐KDR‐IGFBP3 is similar to the typical angiogenic subgroup EC‐KDR‐ESM1,^7^, ^56^ while the expression level of EC‐ACKR1 is weak, suggesting that EC‐ACKR1 may display weak reactivity towards anti‐VEGFR drugs.^57^

In addition, some EC subsets express both VEGFR1 and VEGFR2, through multiple receptors activating downstream signaling responses. These results suggest that drugs targeting single VEGF receptors may not be able to completely block downstream pathways. The compensation of receptor and pathway may be a strategy adopted by ECs to resist TKIS.

Worryingly, IMECs may also be involved in regulating angiogenesis and responding to vascular regulatory signals. The GO analysis suggests the transcriptional activity of angiogenesis genes is upregulated in tumor‐associated IMECs. This upregulation involves VEGFR receptor gene KDR, alongside potential proangiogenic genes such as RHOJ,^35^ ETS1,^36^ and HIF1A.^37^, ^38^ Previous studies have demonstrated that these three latter genes play a significant role in promoting angiogenesis within tumors, thus presenting potential therapeutic targets. Additionally, there are various interaction pathways among ECs, such as the NOTCH signaling pathway^39^, ^40^, ^41^ and the EphA signaling,^42^, ^43^ which are related to the formation of microvessels. IMECs, like the angiogenic subgroup, serve as both signal transmitters and receivers in cell‐cell communication, and EC‐KDR‐IGFBP3 has the highest score. These three subgroups may regulate each other and respond to angiogenic signals, participating in the establishment of a complex angiogenesis regulatory network.

The proportion of EC subsets may be helpful in predicting the therapeutic efficacy of AATs. Bevacizumab is a pioneering targeted medicine and the inaugural angiogenesis inhibitor to receive approval.^3^ In patients who responded to bevacizumab therapy, the proportion of the angiogenic subgroup EC‐KDR‐ESM1 was significantly increased, while the proportion of EC‐KDR‐IGFBP3 was significantly decreased. This suggests that EC‐KDR‐ESM1 is a target subgroup for AATs, while EC‐KDR‐IGFBP3 is associated with therapeutic inefficacy. The reason for the ineffective treatment may be that EC‐KDR‐IGFBP3 has compensatory mechanisms for single‐target drug therapy and the nourishing effect on angiogenic ECs. Depletion of EC‐KDR‐IGFBP3 may be a potential combination therapy for AATs. Interestingly, this study did not observe an effect of EC‐ACKR1 on bevacizumab treatment. The small sample size is a possible explanation. However, it may also be related to the weak expression of FLT and KDR in EC‐ACKR1, which lead to insensitivity to anti‐VEGFR drugs.

Currently, the combination of immunotherapy and AATs is believed to reverse the tumor‐suppressive microenvironment, promote vascular normalization and enhance antitumor immune response.^58^ The combinations of AAT with immunotherapies have demonstrated promising benefits in the treatment of various cancers, encompassing CRC,^59^ thymic carcinomas,^60^ nonsmall cell lung cancer,^61^ and clear‐cell renal‐cell carcinoma.^62^ This study revealed that immunotherapy exerts a significant impact on a subpopulation of ECs within the tumor microenvironment. After immunotherapy, an increase in the proportion of EC‐ACKR1 within tumor tissue was observed, which is consistent with previous reports.^19^ Furthermore, the level of MHC‐II interaction between IMECs and CD4^+^ T cells was augmented, while the angiogenic activity signature score decreased. These observed changes suggest that immunotherapy may exhibit a synergistic effect with AATs, collectively influencing the regulation of ECs. This offers novel insights into the potential application of combined therapeutic strategies.

However, the study has several limitations. First, despite the extensive bioinformatics approach employed in this study, there remains a lack of specific validation for IMEC subsets and cellular functional assays. This limitation hinders our understanding of the precise impact that differential gene expression levels have on cellular phenotypes. Second, while this study identified subsets with an IMECs phenotype in primary CRC, the specific conditions and exact mechanisms that alter immunomodulatory arms of IMECs need to be further explored. The combination of AATs and immunoenhancement therapy has been shown to improve prognosis.^63^ Whether this is also related to reshaping the phenotype of IMECs remains to be investigated. Moreover, the specific crosstalk mechanism and extent in vascular‐associated signaling pathways remain to be fully characterized. Finally, the bevacizumab therapeutic dataset is relatively small and the limited proportion of EC subsets within the tumor may introduce additional sources of error into the deconvolution process. Therefore, it is necessary to prospectively collect larger datasets or validate using tissue‐specific knockout techniques.

## AUTHOR CONTRIBUTIONS

**Jingyi Wen**: Conceptualization, data curation, formal analysis, investigation, methodology, project administration, resources, software, supervision, validation, visualization, writing—original draft, writing—review & editing.

## CONFLICT OF INTEREST STATEMENT

The authors declare no conflicts of interest.

## Supporting information

Supporting information.

Supporting information.

Supporting information.

Supporting information.

Supporting information.

## Data Availability

Our study is based on open source data, so there are no ethical issues and other conflicts of interest. The datasets [GSE178341] for this study can be found in the [GEO] [https://www.ncbi.nlm.nih.gov/geo/query/acc.cgi?acc=GSE178341]. The datasets [GSE188711] for this study can be found in the [GEO] [https://www.ncbi.nlm.nih.gov/geo/query/acc.cgi?acc=GSE188711]. The datasets [GSE132465] for this study can be found in the [GEO] [https://www.ncbi.nlm.nih.gov/geo/query/acc.cgi?acc=GSE132465]. The datasets [GSE144735] for this study can be found in the [GEO] [https://www.ncbi.nlm.nih.gov/geo/query/acc.cgi?acc=GSE144735]. The datasets [GSE19862] for this study can be found in the [GEO] [https://www.ncbi.nlm.nih.gov/geo/query/acc.cgi?acc=GSE19862]. The datasets [GSE19860] for this study can be found in the [GEO] [https://www.ncbi.nlm.nih.gov/geo/query/acc.cgi?acc=GSE19860]. The datasets [GSE205506] for this study can be found in the [GEO] [https://www.ncbi.nlm.nih.gov/geo/query/acc.cgi?acc=GSE205506]. The detailed code is available from the link of GitHub (https://github.com/wenjingyi-1/001-IMECs).
