# Supplementary material for: Single‐cell transcriptomics reveals antigen‐presenting capacity and therapeutic resistance potential of immunomodulatory endothelial cells in colorectal cancer
Source: Immun Inflamm Dis. 2024 Jun 14;12(6):e1311. doi: 10.1002/iid3.1311 (PMC11177288; doi:10.1002/iid3.1311)
Supplement: Supplementary file 1 — Supporting information. [file IID3-12-e1311-s002.docx]

# Supplementary Figures

**Supplementary Figure S1.** Detailed clustering basis. (A) Dotplot shows the expression of selected marker genes for the first cluster within tumor tissue. (B) Dotplot depicting the expression of selected marker genes in mural and smooth muscle cells within tumor tissue. (C) Dotplot depicting the expression of selected marker genes in fibroblasts within tumor tissue. (D) UMAP clustering of normal stromal cells. (E) Dotplot depicting the expression of selected marker genes in mural and smooth muscle cells within adjacent normal tissue. (F) Dotplot depicting the expression of selected marker genes in fibroblasts within adjacent normal tissue. (G) Dotplot depicting the expression of selected marker genes in CD4^+^ T cells within tumor tissue.

**Supplementary Figure S2.** Assessment of dissociation score gene signature in different EC subpopulations. (A) immediate early genes scores (left) and heat shock proteins scores (right) in the original dataset. (B) immediate early genes scores (left) and heat shock proteins scores (right) in the validation dataset.

**Supplementary Figure S3.** Detailed clustering basis in the validation dataset. (A) UMAP clustering of stromal cells. (B) Dotplot depicting the expression of selected marker genes in stromal cells. (C) Dotplot depicting the expression of selected marker genes in antigen-experienced CD4+ T cells.

**Supplementary Figure S4.** GO analysis of EC-KDR-ESM1 and EC-STMN1, shown from top to bottom as MF, CC and BP. (A) Results of EC-KDR-ESM1. (B) results of EC-STMN1.

**Supplementary Figure S5.** GO analysis of transcriptional activity changes of EC-ACKR1, shown from top to bottom as MF, CC and BP. The biological functions of interest have been outlined, where antigen presentation is represented by the green line and angiogenesis regulation by the red line. (A) The up-regulated transcriptional activity in tumor tissues. (B) The down-regulated transcriptional activity in tumor tissues.

**Supplementary Figure S6.** GO analysis of transcriptional activity changes of EC-KDR-IGFBP3 (EC-IGFBP3), shown from top to bottom as MF, CC and BP. The biological functions of interest have been outlined, where antigen presentation is represented by the green line and angiogenesis regulation by the red line. (A) The up-regulated transcriptional activity in tumor tissues. (B) The down-regulated transcriptional activity in tumor tissues.

# Supplementary Tables

**Supplementary Table 1.** Differential genes for each subset in the first cluster of tumor tissue.

**Supplementary Table 2.** Differential genes for each subset in the cluster of stromal cells.

**Supplementary Table 3.** Differential genes for GO analysis of EC-ACKR1.

**Supplementary Table 4.** Differential genes for GO analysis of EC-KDR-ESM1.

**Supplementary Table 5.** Differential genes for GO analysis of EC-KDR-IGFBP3.

**Supplementary Table 6.** Differential genes for GO analysis of EC-STMN1.

**Supplementary Table 7.** The full Table showing the differentially up/down regulated genes for the two IMEC clusters

**Supplementary Table 8.** Signature matrix file and the gene signatures
