# Supplementary figures and images for: Single‐cell transcriptomics reveals antigen‐presenting capacity and therapeutic resistance potential of immunomodulatory endothelial cells in colorectal cancer
Source: Immun Inflamm Dis. 2024 Jun 14;12(6):e1311. doi: 10.1002/iid3.1311 (PMC11177288; doi:10.1002/iid3.1311)

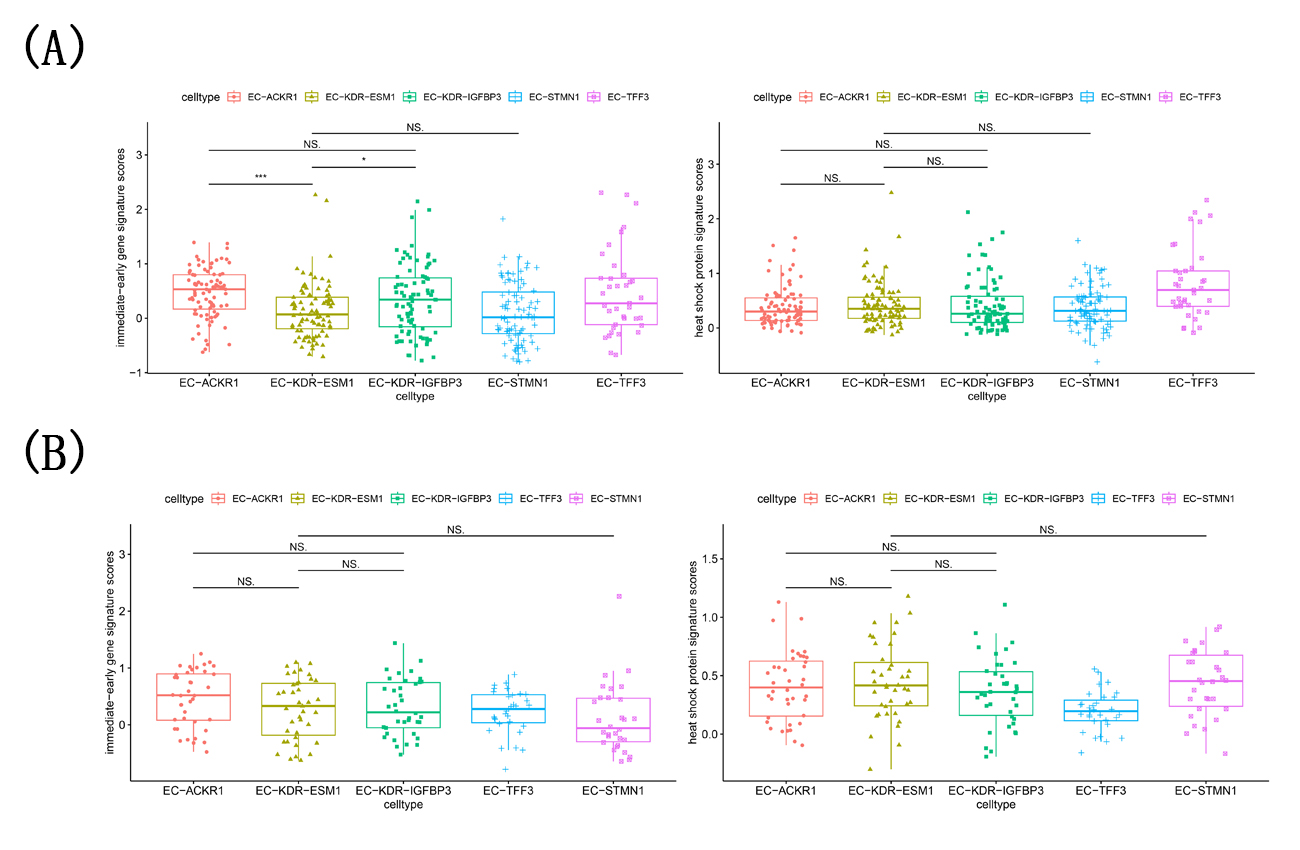

Supplement: Supplementary file 2 — Supporting information. [file IID3-12-e1311-s003.zip › S2.jpg]

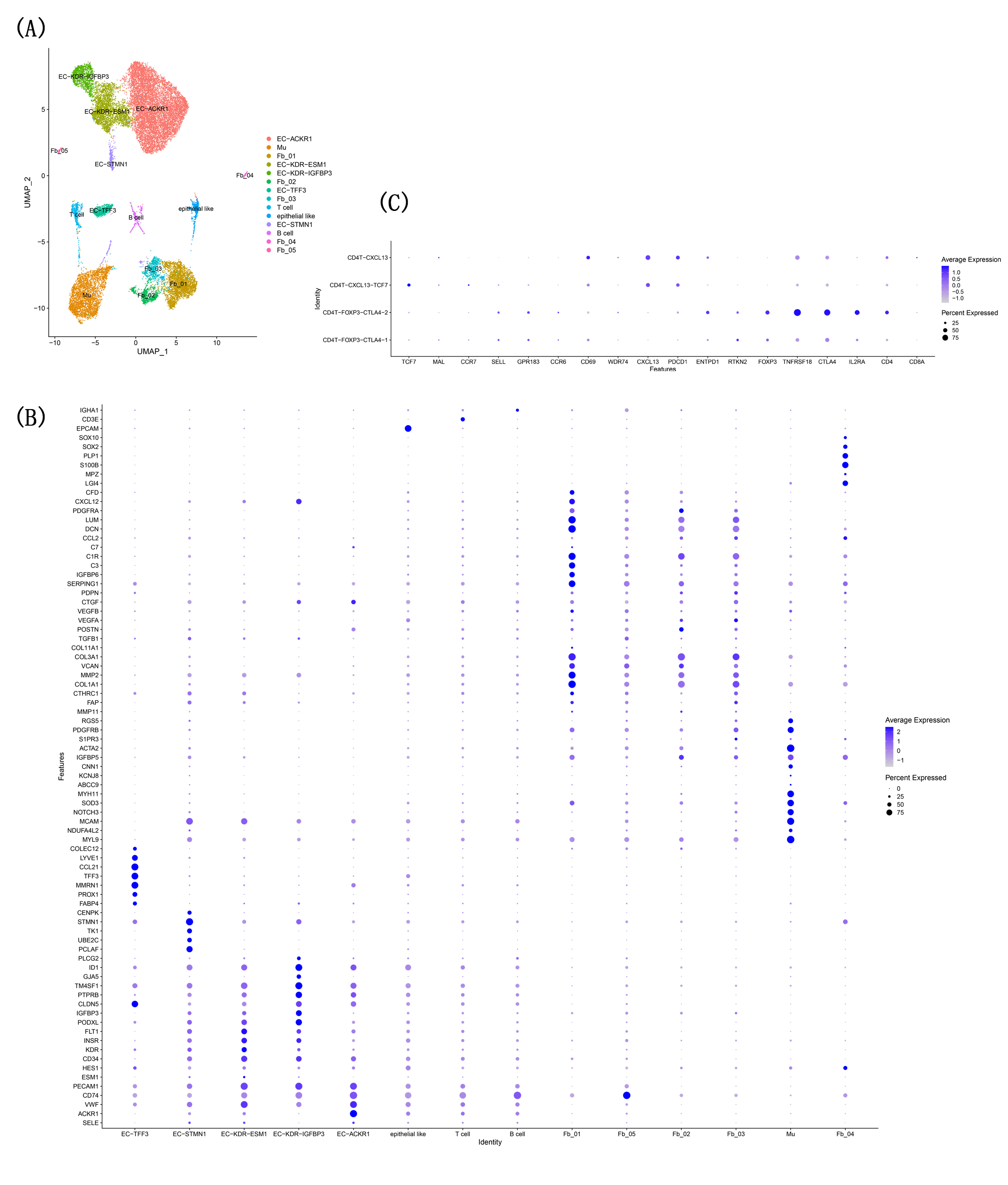

Supplement: Supplementary file 2 — Supporting information. [file IID3-12-e1311-s003.zip › S3.jpg]

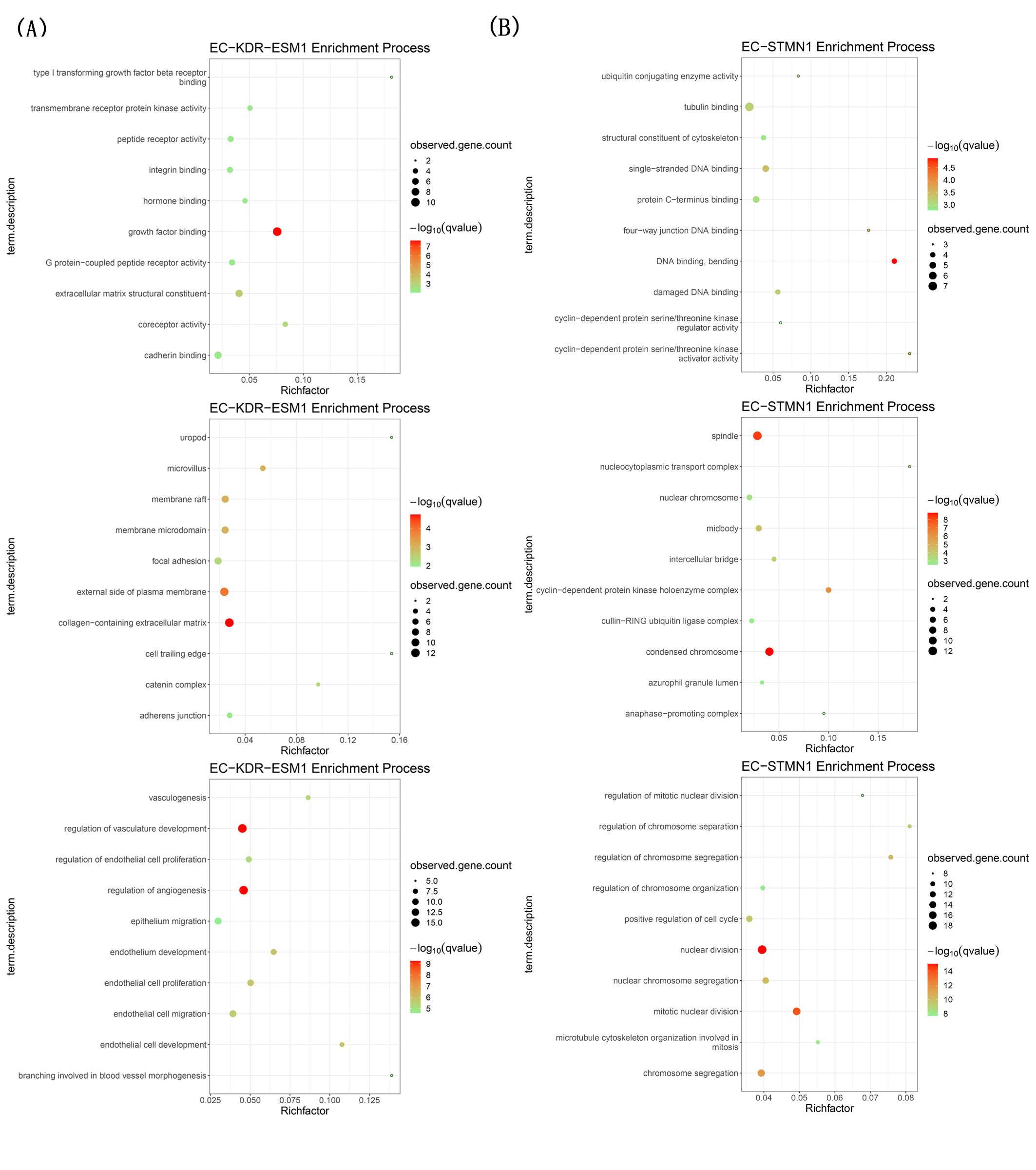

Supplement: Supplementary file 2 — Supporting information. [file IID3-12-e1311-s003.zip › S4.jpg]

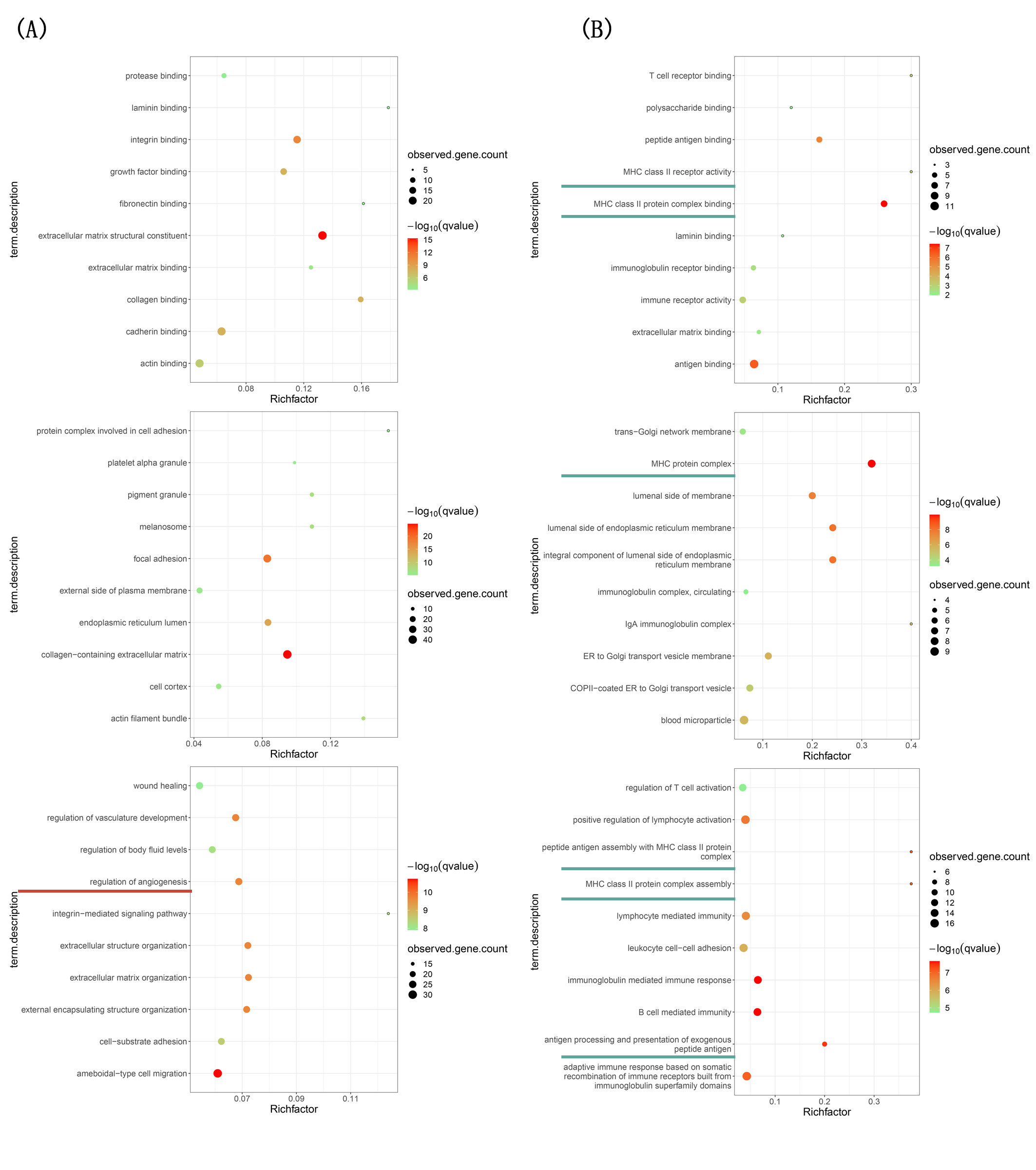

Supplement: Supplementary file 2 — Supporting information. [file IID3-12-e1311-s003.zip › S5.jpg]

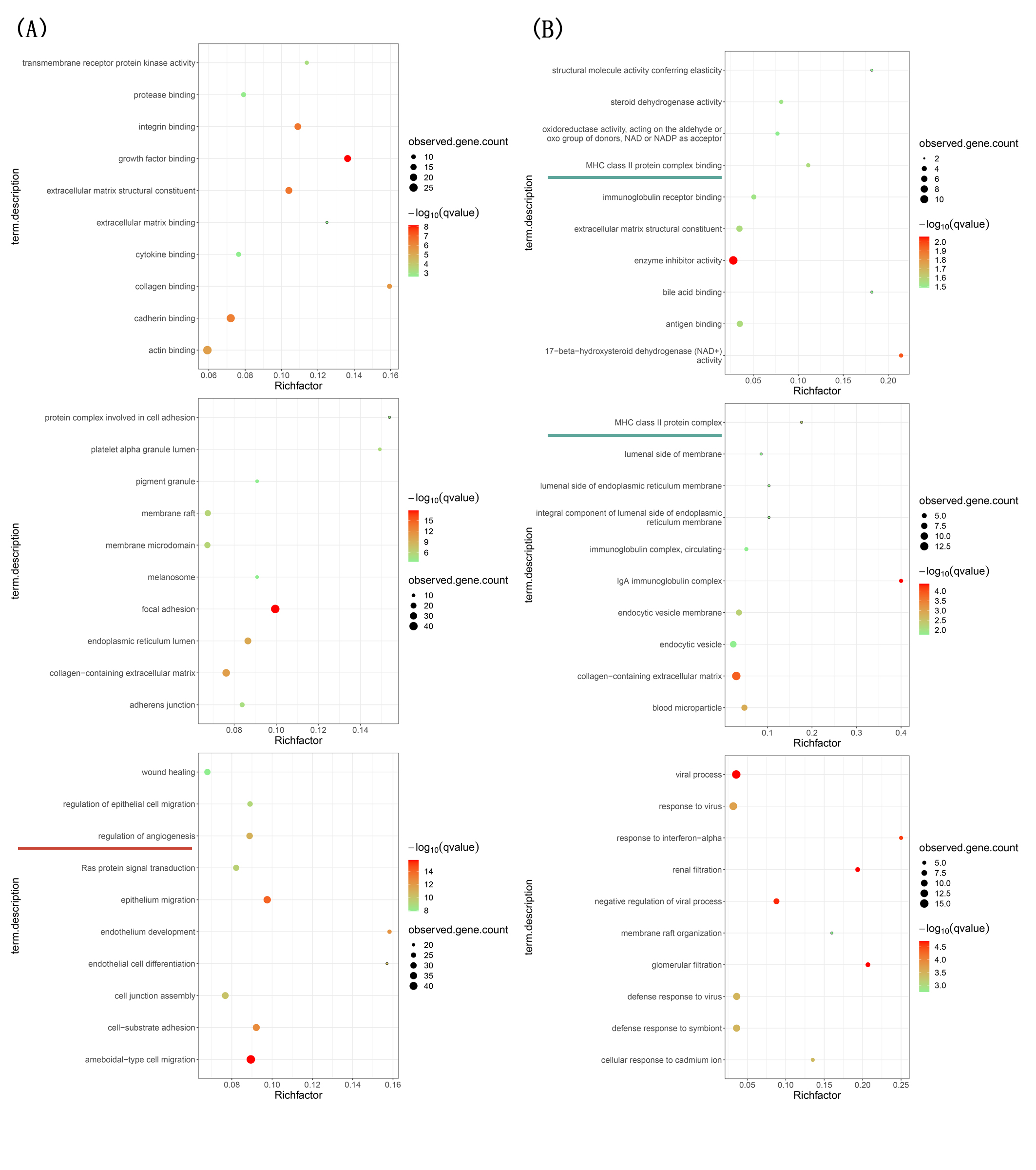

Supplement: Supplementary file 2 — Supporting information. [file IID3-12-e1311-s003.zip › S6.jpg]

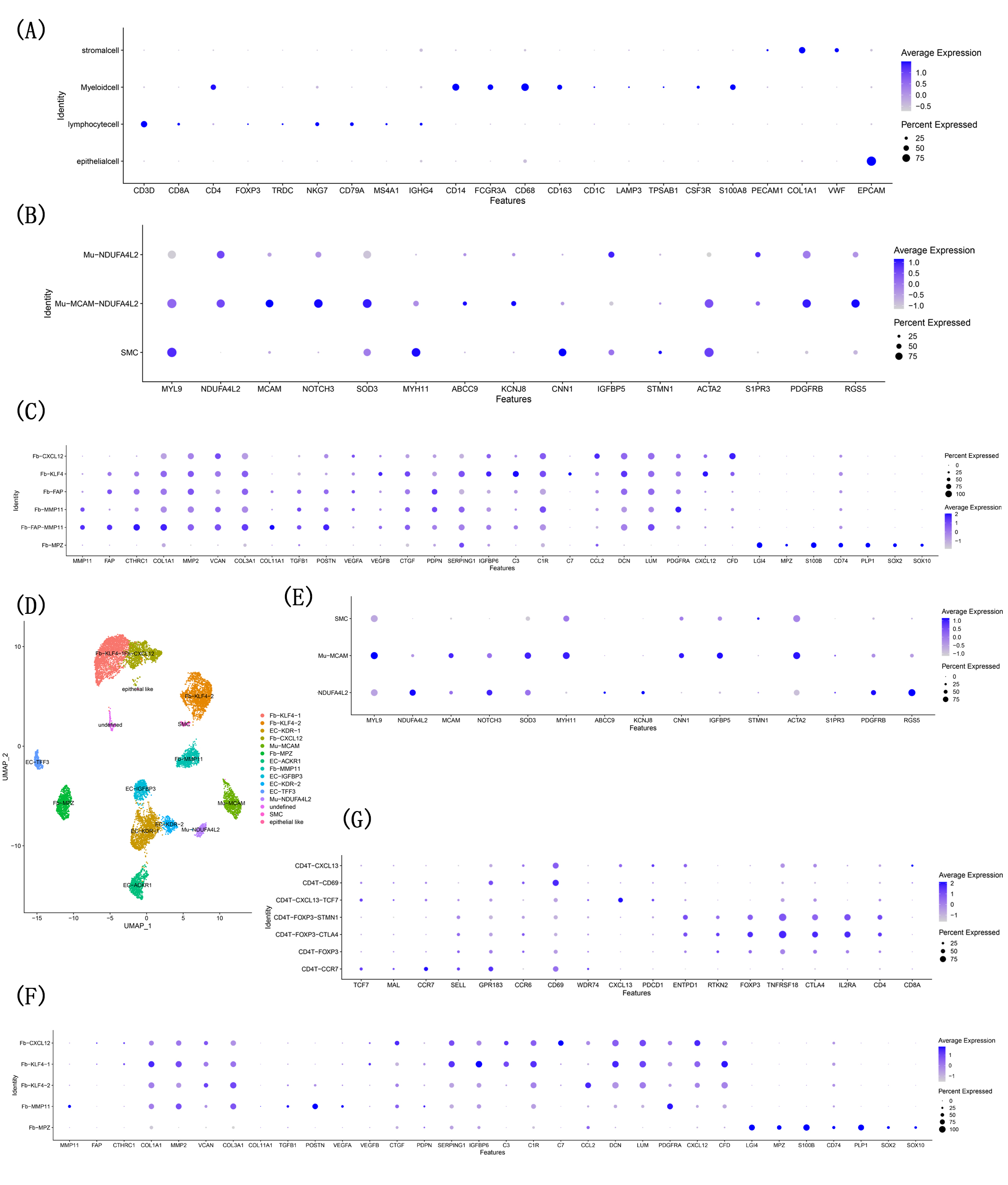

Supplement: Supplementary file 2 — Supporting information. [file IID3-12-e1311-s003.zip › S1.jpg]

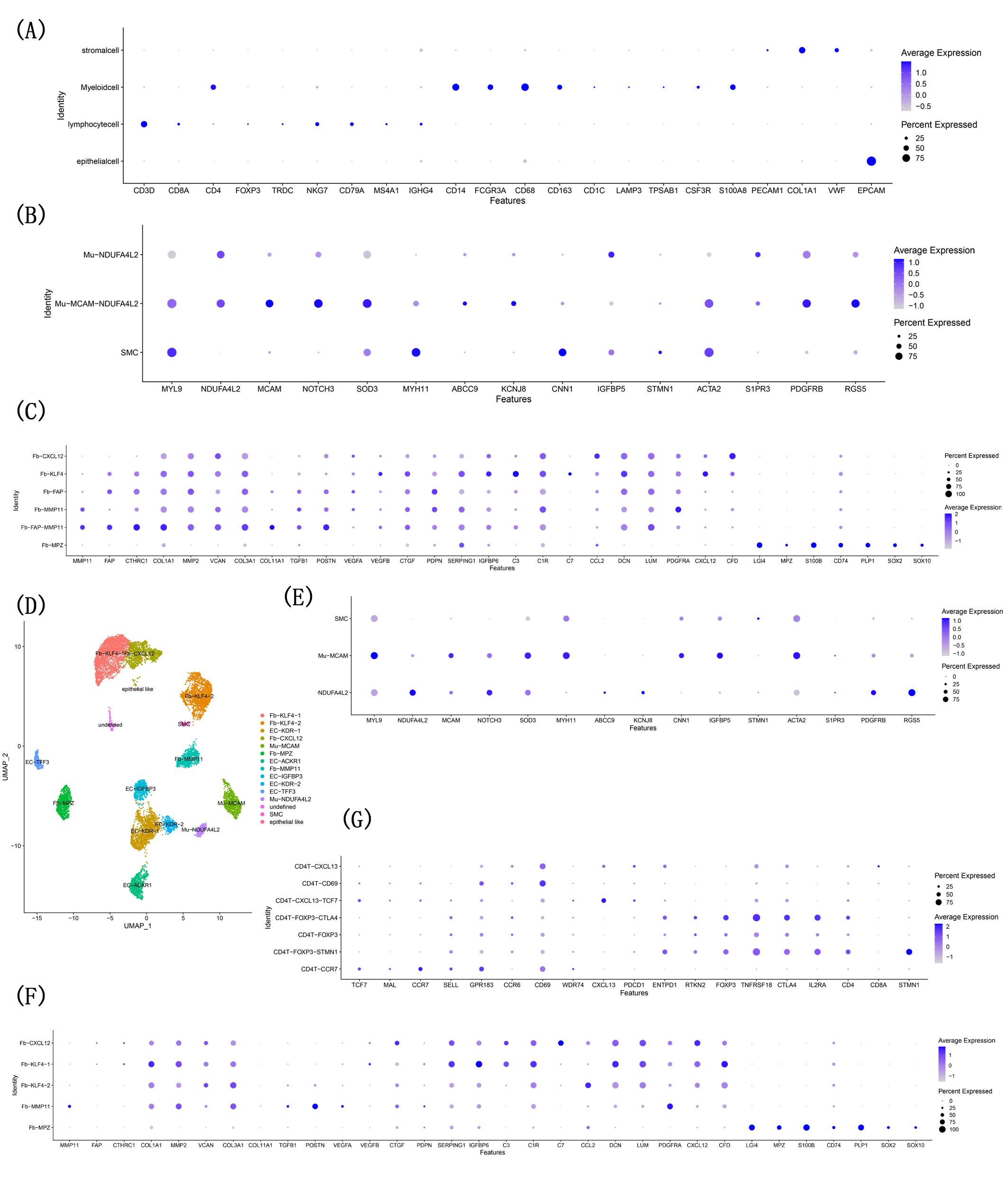

Supplement: Supplementary file 4 — Supporting information. [file IID3-12-e1311-s004.jpg]

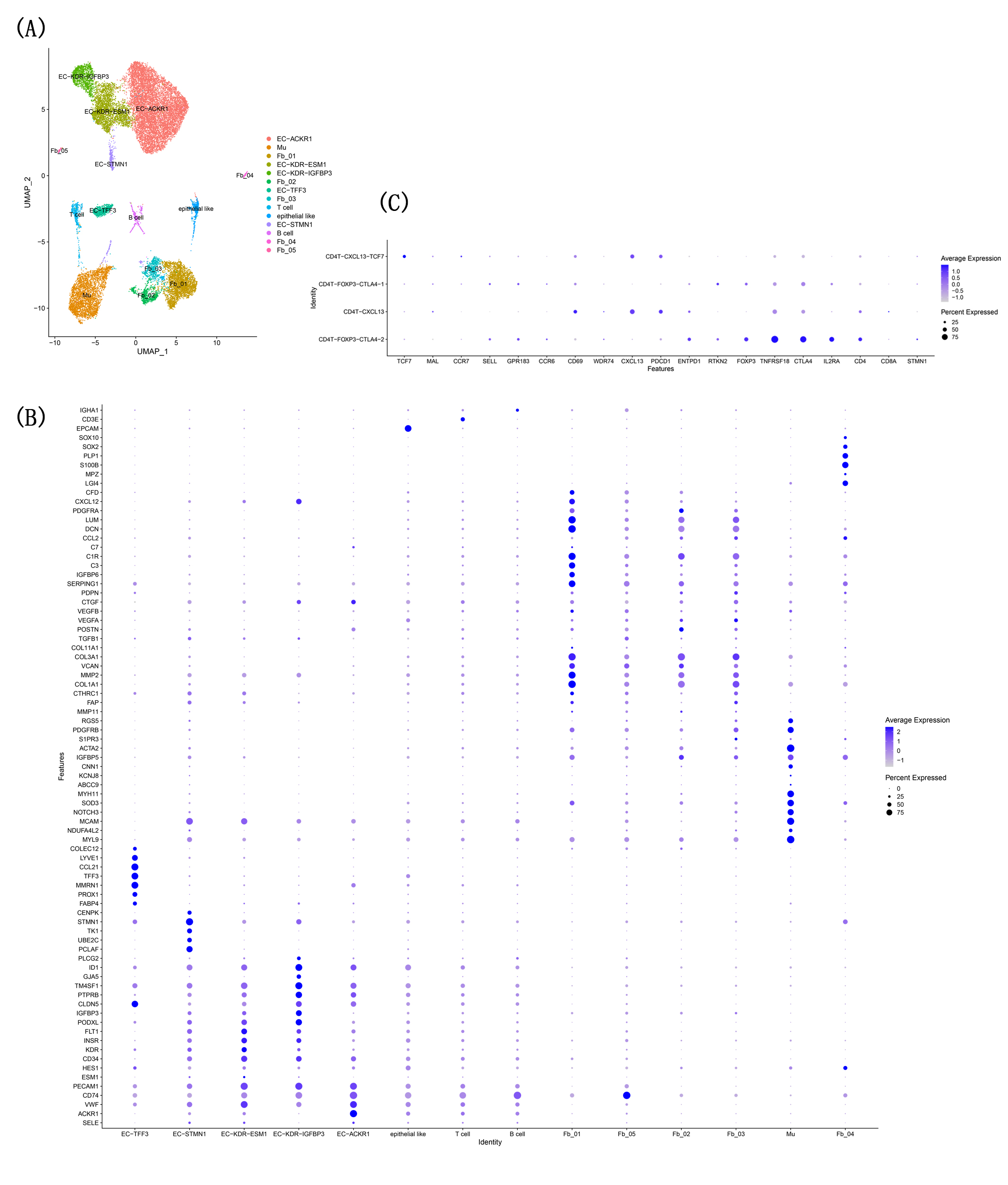

Supplement: Supplementary file 5 — Supporting information. [file IID3-12-e1311-s001.jpg]
